# Tuning the Emission of Cationic Iridium (III) Complexes Towards the Red Through Methoxy Substitution of the Cyclometalating Ligand

**DOI:** 10.1038/srep12325

**Published:** 2015-07-16

**Authors:** Kamrul Hasan, Ashu K. Bansal, Ifor D.W. Samuel, Cristina Roldán-Carmona, Henk J. Bolink, Eli Zysman-Colman

**Affiliations:** 1 Département de Chimie, Université de Sherbrooke, 2500 Boul. de l’Université, Sherbrooke, QC, Canada, J1K 2R1; 2 Organic Semiconductor Centre, SUPA, School of Physics and Astronomy, University of St. Andrews, North Haugh, St. Andrews, Fife, UK, KY16 9SS; 3 Instituto de Ciencia Molecular, Universidad de Valencia, C/ Catedrático J. Beltrán 2, 46980 Paterna (Valencia), Spain; 4 Department of Physical Chemistry and Applied Thermodynamics, Campus Rabanales, Ed. C3, University of Cordoba, 14014, Spain; 5 Organic Semiconductor Centre, EaStCHEM School of Chemistry, University of St Andrews, St Andrews, Fife, KY16 9ST, UK

## Abstract

The synthesis, characterization and evaluation in solid-state devices of a series of 8 cationic iridium complexes bearing different numbers of methoxy groups on the cyclometallating ligands are reported. The optoelectronic characterization showed a dramatic red shift in the absorption and the emission and a reduction of the electrochemical gap of the complexes when a methoxy group was introduced *para* to the Ir-C bond. The addition of a second or third methoxy group did not lead to a significant further red shift in these spectra. Emission maxima over the series ranged from 595 to 730 nm. All complexes possessing a motif with a methoxy group at the 3-position of the cyclometalating ligands showed very short emission lifetimes and poor photoluminescence quantum yields whereas complexes having a methoxy group at the 4-position were slightly blue shifted compared to the unsubstituted parent complexes, resulting from the inductively electron withdrawing nature of this directing group on the Ir-C bond. Light-emitting electrochemical cells were fabricated and evaluated. These deep red emitters generally showed poor performance with electroluminescence mirroring photoluminescence. DFT calculations accurately modelled the observed photophysical and electrochemical behavior of the complexes and point to an emission from a mixed charge transfer state.

Light Emitting Electrochemical Cells (LEECs)^1^,^2^ are solid state lighting devices that typically incorporate a charged phosphorescent Ionic Transition Metal Complex (iTMC) as the luminophore. These devices offer some advantages over organic light-emitting diodes (OLEDs)^2^. LEECs have simple device architectures (single- or two-layer devices), use air-stable high work function electrodes (*e.g.*, Al, Au) and are fabricated using solution printing processing with benign solvents such as acetonitrile (ACN), thus giving the potential to operate without encapsulation. They are thus cheaper to manufacture and can be driven by alternating current (AC). Employing LEEC technology, large-area artificial illumination is thus easier to attain^3^,^4^. In LEECs the iTMC accomplishes several functions including: lowering of the charge injection barrier by the displacement of ions; facilitating charge transport through iterative redox cycling; and generating light through phosphorescence. Based on the similarities in mechanism, LEECs should be able to attain similar efficiencies to OLEDs. Despite the advantages listed above, LEECs incorporating iTMCs have several weaknesses: (i) slow turn-on time compared to OLEDs; (ii) low EQEs; (iii) limited stability of the device; and (iv) color quality^5^. Red light emission is important as a component of white light emitting devices.

The principal strategies utilized to date to tune the emission energy into the red (λ_em_ > 630 nm) include the use of highly conjugated ancillary ligands^6^,^7^,^8^,^9^,^10^,^11^,^12^,^13^,^14^,^15^,^16^,^17^,^18^,^19^,^20^,^21^,^22^ (N^N) such as biquinolines or the incorporation of electron-withdrawing groups^23^,^24^ onto the backbone of the ancillary ligand. Likewise, using conjugated cyclometallating ligands (C^N) such as phenylquinolines has also been exploited as a strategy for producing red-light emission^10^,^16^,^20^,^25^,^26^,^27^,^28^. Red-light emission was also obtained through energy transfer to a covalently tethered organic perylenediimide^29^. The use of pyridyl oxadiazoles^30^ and dipyridyl pyrazines^31^ as ancillary ligands has also resulted in deep red emission. LEECs incorporating these complexes emit with electroluminescent (EL) λ_max_ between 630–665 nm and peak EQEs of between 0.02–7.4%^32^.

Oddly, the use of electron donating groups on the phenyl moiety of the C^N ligand has not be explored as a strategy to tune the emission energy to the red in cationic iridium(III) complexes despite the common use of electron withdrawing fluorine atoms to push the emission to the blue^9^,^33^,^34^,^35^,^36^,^37^,^38^,^39^. Herein we systematically explore the impact of methoxy substitution on the phenyl ring of the C^N ligands on the optoelectronic properties of a series of eight cationic iridium complexes^40^,^41^. DFT calculations rationalize the observed behavior. LEECs fabricated with these emitters demonstrate that this is a viable strategy for emission tuning in electroluminescent devices to the red, leading to a maximum emission wavelength of 650 nm and CIE_(x,y)_ coordinates (0.61, 0.38) for the reddest complex. Unfortunately, the performance suffers in terms of brightness and device stability.

## Results and Discussion

### Synthesis

**Figure 1:**
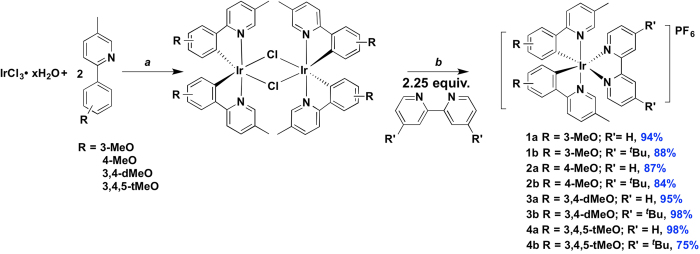
Synthesis of complexes in study. Reagents and conditions: ^*a*^2-EtOC_2_H_4_OH/H_2_O (8:1 v/v), 110 °C, N_2_, 19 h. ^*b*^i. CH_2_Cl_2_/MeOH (1:1 v/v), 55 °C, 19 h, N_2_; ii. Excess aq. NH_4_PF_6_.

The targeted methoxy-substituted C^N ligands were synthesized in two steps from the corresponding aryl bromide via arylboration and Suzuki coupling with 2-bromo-5-methyl pyridine (Figure S23). The non-optimized yields for boronic acid formation ranged from 36–65% while the cross-coupling reaction proceeded nearly quantitatively. The iridium dimers, [(C^N)_2_Ir(Cl)]_2_, were synthesized following the procedure first described by Nonoyama^42^. Heteroleptic cationic complexes **1a-4b** were obtained in excellent yield through the cleavage of these dimers with 2.25 equiv. of ancillary diimine ligand (2,2’-bipyridine, bpy, or 4,4’-di*tert-*butyl-2,2’-bipyridine, d*t*Bubpy) in refluxing DCM/MeOH (1:1 v/v) under N_2_ (Fig. 1)^19^. The complexes were isolated as their hexafluorophosphate salts following the dropwise addition of a methanolic solution of the corresponding chloride salts into an aqueous solution of NH_4_PF_6_. Each complex was purified by recrystallization from a 1:1 solution of DCM and di*iso*propyl ether. The structural identity and purity of each of the complexes were ascertained through ^1^H NMR and ^13^C NMR spectroscopy (Figures S1-S22), ESI-HRMS and melting point analyses.

**Figure 2:**
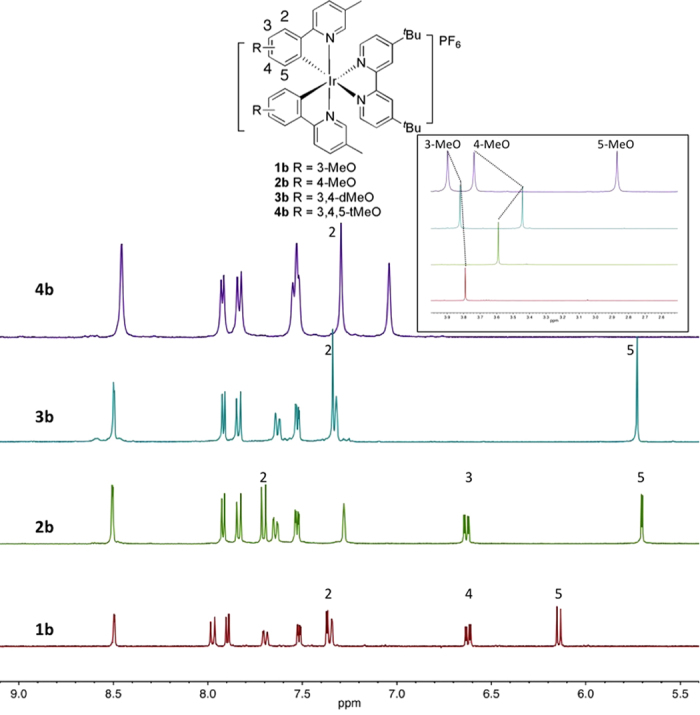
Aromatic region of the ^1^H NMR spectra of 1b-4b in CD_3_CN at 298 K. Inset shows MeO region of spectra.

The aromatic regions of the ^1^H NMR spectra of **1b, 2b, 3b** and **4b** obtained in CD_3_CN are shown in Fig. 2. From the overall pattern of the ^1^H NMR it is evident that each of **1b-4b** possesses similar *C*_2_-symmetry. By contrast, Davies and co-workers reported the presence of two isomers for the related [Ir(3-MeOppz)_2_(bpy)]PF_6_ wherein a second minor isomer was also detected when cyclometallation occurred at C_2_ (3-MeOppzH = *N*-(3-methoxyphenyl)pyrazole)^40^. As previously observed by Davies, the H_5_ signal is the most upfield proton and is most strongly influenced by the adjacent 4-MeO substituent. The chemical shifts of the d*t*Bubpy ligand (the three most downfield signals) are essentially invariant to methoxy substitution on the C^N ligands though are shifted slightly downfield in **1b** compared to the other three complexes in the family. The 3-MeO group exhibits the strongest influence on the chemical shift of the H_2_ proton; in its absence, H_2_ in **2b** is situated downfield at 7.71 ppm. The presence of two methoxy groups in **3b** results in a downfield shift of the 3-MeO group and an upfield shift of the 4-MeO group compared to **1b** and **2b**, respectively. However, the additional incorporation of the 5-MeO group, positioned upfield at 2.87 ppm, in **4b** promotes a significant 0.30 ppm downfield shift of the 4-MeO signal compared to that in **3b**.

### Cyclic voltammetry

**Figure 3:**
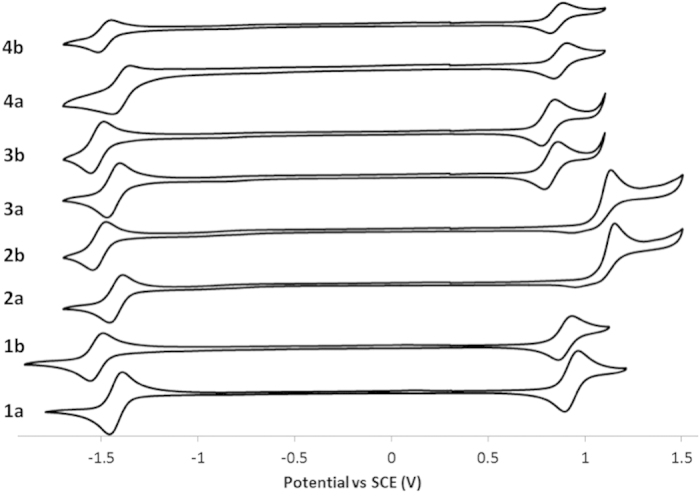
Cyclic voltammograms for 1a-4b recorded at 298 K at 50 mVs^−1^ in deaerated ACN with 0.1 M (*n-*Bu_4_N)PF_6_.

The electrochemical behavior of **1a-4b** was investigated through cyclic voltammetry (CV) studies in deaerated ACN solution containing *n-*NBu_4_PF_6_ as the supporting electrolyte and using Fc/Fc^+^ as an internal standard at 298 K. All potentials are referenced to SCE (Fc/Fc^+^ = 0.38 V in ACN)^43^. The electrochemistry data, obtained at a scan rate of 50 mV s^−1^, are summarized in Table S1. The CV behavior was reproducible at the faster scan rate of 200 mV s^−1^. The CV traces for **1a-4b** are shown in Fig. 3. All complexes exhibit reversible first reduction waves and all complexes save **2a** and **2b** exhibit reversible first oxidation waves.

The first oxidation wave, corresponding to the HOMO, is attributed to the Ir^III^/Ir^IV^ couple with contribution from the C^N ligands. For all the complexes in the study this wave is significantly cathodically shifted compared to both [Ir(ppy)_2_(bpy)]PF_6_ (E_1/2,ox_ = 1.25 V) and [Ir(ppy)_2_(d*t*Bubpy)]PF_6_ (E_1/2,ox_ = 1.29 V) owing to the mesomeric electron donating character of the methoxy group^44^. The incorporation of electron-donating *tert*-butyl groups generally promote a further cathodic shift of between 0.02–0.06 V. Methoxy groups are Janus-like, being electron donating mesomerically but electron withdrawing inductively, evident from an evaluation of Hammet sigma parameters (σ_m_ = 0.12, σ_p_ = −0.27). Complexes **2a** and **2b**, with 4-MeOppy C^N ligands, are shifted cathodically the least in the series compared to their unsubstituted analogs. Moving the MeO group to the 3-position results in a 200 mV shift to lower positive potentials for **1a** vs **2a** and 230 mV for **1b** vs **2b**. The presence of two MeO groups results in a synergistic destabilization of the oxidation wave by a further 110 mV for **3a** vs **1a** and 120 mV for **3b** vs **1b**. The addition of a third MeO group in **4a** and **4b** does not result in any further appreciable tuning of the oxidation wave. This first oxidation wave is irreversible for **2a** and **2b** but is a pseudo-reversible one-electron process for the other complexes in the series.

A second irreversible oxidation wave was found between 1.30–1.57 V in all eight complexes. This oxidation wave is the least cathodically shifted in **2a** and **2b** with peak potentials of 1.55 and 1.57 V, respectively. By contrast, for **1a** and **1b** the second oxidation waves at 1.33 and 1.32 V, respectively, are significantly cathodically shifted compared to those in **2a** and **2b**. These second oxidation waves are pushed to lower positive potentials with the presence of two methoxy groups with irreversible oxidation waves for **3a** and **3b** of 1.13 and 1.25 V, respectively. With three methoxy groups present in the cases of **4a** and **4b** these waves remain at 1.21 V. From the similar electrochemical behavior between the first and second oxidation waves, we can conclude that this second wave is also localized on the C^N ligand.

The reduction potential for **1a-4a** is quite invariant at between −1.40 and −1.42 V; similarly that for **1b-4b** resides between −1.49 and −1.52 V. The clustering of reduction potentials points to a reversible one-electron first reduction wave, and thus the LUMO, that is localized on the ancillary diimine ligand. The presence of the MeO groups though located on the C^N ligands act to destabilize the reduction wave compared to both [Ir(ppy)_2_(bpy)]PF_6_ (E_1/2,red_ = −1.36 V) and [Ir(ppy)_2_(d*t*Bubpy)]PF_6_ (E_1/2,red_ = −1.38 V). Surprisingly, there is a significantly larger influence for the family of complexes containing a d*t*Bubpy N^N ligand than the congeners **1a-4a**.

The electrochemical gaps for **1a-4b** range from 2.27 for **3a** to 2.63 V for **2b**. Thus, the use of a 4-MeOppy C^N ligand has a negligible effect on the tuning of the HOMO-LUMO gap compared to the parent complexes [Ir(ppy)_2_(bpy)]PF_6_ (ΔE_redox_ = 2.65 V) and [Ir(ppy)_2_(d*t*Bubpy)]PF_6_ (ΔE_redox_ = 2.71 V). The use of d*t*Bubpy as the ancillary ligand promotes a widening of the gap by between 0.08–0.11 V except for **2a** and **2b** where there is little influence observed. Within each family of complexes **3a** and **3b** possess the smallest electrochemical gaps of 2.27 and 2.35 V, respectively.

### UV-Visible absorption spectroscopy

**Figure 4:**
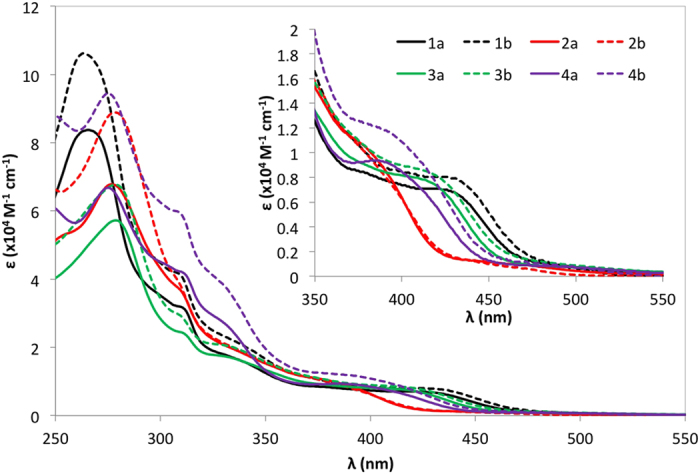
UV-vis absorption spectra for 1a-4b in ACN at 298 K. Inset: Zoomed UV-vis spectra for the low energy region between 350–550 nm.

The UV-Visible absorption spectra for **1a-4b** were recorded in aerated ACN at 298 K, the spectra of which are shown in Fig. 4. The calculated molar absorptivities, ε, are collected in Table S2. As with many cationic heteroleptic iridium complexes the absorption spectra exhibit certain characteristic features. There are intense bands between 244 and 278 nm, which are assigned to the spin-allowed ligand centered (^1^LC) π-π* transitions for cyclometallating (C^N) ppy and ancillary (N^N) bpy ligands. Lower intensity absorption bands below 300 nm are assigned to spin-allowed mixed charge transfer (CT) bands while bands visible past 450 nm are assigned to spin-forbidden mixed CT bands.

The molar absorptivities for each of the absorption bands are on the order of 10^4^ M^−1^ cm^−1^. The profile and intensity of these bands are similar to other bis(heteroleptic) cationic iridium complexes reported elsewhere in the literature^45^,^46^,^47^,^48^,^49^. The ^1^LC bands for the d*t*Bubpy family of complexes are generally about 20% more intense than those of the bpy family of complexes. The ^1^LC bands for **1a** and **1b**, found at ca. 265 nm, are hypsochromically shifted by 1635 and 2052 cm^−1^, respectively, compared to those of **2a** and **2b** found at 277 nm. The absorption spectra for the latter two complexes also show the presence of an addition ^1^LC band at 255 and 250 nm, respectively. This absorption profile is mirrored for **3a**, **3b**, **4a** and **4b** wherein a high intensity band for each is located between 275–279 nm and the lower intensity higher energy band is also observable in **4a** and **4b** (244 and 248 nm, respectively); this latter band appears only as a shoulder in **3a** and **3b**. All complexes possess an absorption band at ca. 308–312 nm. Similar to other heteroleptic iridium (III) cationic complexes^50^,^51^, hypochromic bands between 330–425 nm result from mixed (^1^CT) transitions resulting from a combination of metal-to-ligand and ligand-to-ligand CT transitions (^1^MLCT and ^1^LLCT, respectively). The low energy bands for **1a** and **1b** are located at 425 nm whereas **2a** and **2b** these are found at 390 nm. The red shifted nature of these transitions in **1a** and **1b** are in line with their electrochemistry and smaller electrochemical gaps. Additionally, owing to strong spin-orbit coupling on iridium there is the presence of very low intensity bands in the region of 400–500 nm that correspond to spin-forbidden ^3^CT transitions.

From the UV-Vis spectroscopy we can conclude that placing a methoxy group in the 3-position, *para* to the C-Ir bond, invokes a larger red-shift in the absorption compared to substitution at the 4-position. The incorporation of two methoxy groups does not lead to a further red shift in the absorption profile. In fact, **3a** and **3b** possess bands at 419 and 424 nm, respectively that are slightly blue shifted compared to those of **1a** and **1b**. With three methoxy groups present on the C^N ligand, a further blue shift is imparted in **4a** and **4b** where these transitions are found at 385 and 395 nm, respectively.

### Solution state photophysical behavior

**Figure 5:**
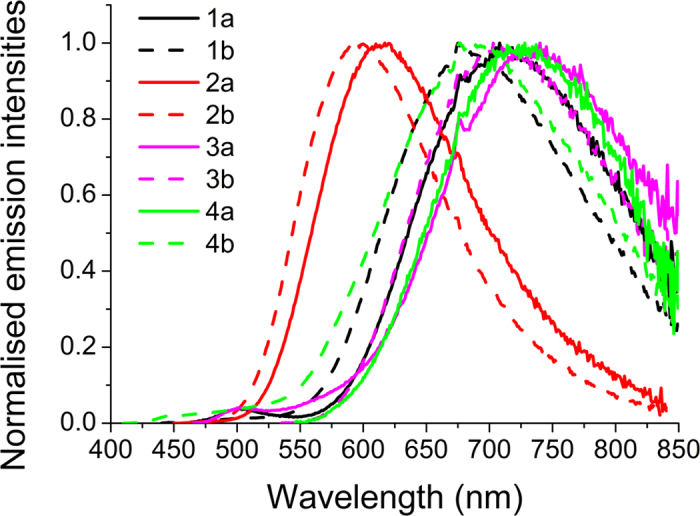
Steady state emission spectra for 1a-4b in degassed ACN at 298 K.

The steady state emission spectra for complexes **1a**-**4b** were recorded in degassed ACN at 298 K (Fig. 5). The emission profiles are broad and featureless with emission maxima ranging from 580–730 nm. The yellow-to-near infrared emission is assigned to a mixed metal-to-ligand and ligand-to-ligand charge transfer transitions (^3^MLCT and ^3^LLCT), commonly observed for heteroleptic cationic iridium complexes^9^,^49^. Excepting **2a** and **2b**, the emission bands of these complexes are red shifted compared to [Ir(ppy)_2_ (bpy)]PF_6_ (λ_em_ = 610 nm), which had been previously reported by us^49^ and others^52^,^53^,^54^. The emission maxima for **3a**, **3b**, **4a** and **4b** are further red-shifted compared to complexes **1a** and **1b**. The reddest emission occurs in **3a**. The trends observed in the emission spectra mirror those in the absorption spectra and the CV thus demonstrating that the *T*_1_ state is modulated similarly to the *S*_0_ ground state. Not surprisingly, the use of the inductively electron-donating d*t*Bubpy N^N ligands in **1b-4b** blue shifts the emission compared to their respective analogs in **1a-4a**.

**Figure 6:**
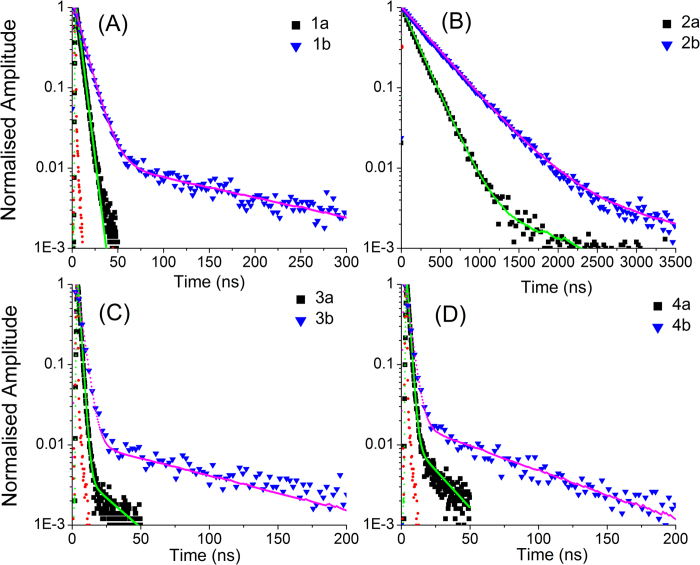
Time-resolved photoluminescence spectra for **1a-4b** in degassed ACN at 298 K. The samples were excited at 390 nm. The emission is collected in each case at the peak of the PL spectra. The red dotted sprectrum represent the instrument response function.

The photoluminescence quantum yields (Φ_PL_) for **1a-4b** were determined in degassed ACN at 298 K using by comparing with a reference standard [Ru(bpy)_3_](PF_6_)_2_ (Ф_PL_ = 9.5%)^55^ under optically dilute conditions as described by Demas and Crosby^56^ (Table 1). With the exception of **2a** (5.7%) and **2b** (15.4%), the Φ_PL_ values are exceedingly low, especially compared to other iridium complexes emitting in the same spectral region^57^. Further information about the photophysics of the compounds were obtained through time-resolved phosphorescence (PL) measurements by exciting the deaerated samples at room temperature at 390 nm. The results are shown in Fig. 6. Compound **1a** shows monoexponential decay with a lifetime of 4.7 ns with no presence of long lived emission.

The presence of the bulky d*t*Bubpy N^N ligand in compound **1b** enhances the lifetime corresponding to phosphorescence emission but still nonradiative decay dominates as evident from poor PLQY and the short-lived decay seen in Fig. 6a. For compounds **2a** and **2b**, Fig. 6b, emission lifetimes are much longer and in the sub-microsecond regime, 194 and 388 ns, respectively, with pre-exponential factors of up to 97%. So the position of the methoxy group strongly influences the photophysical properties of the compounds as we saw with PLQY results. The addition of a further MeO moiety in **3a** drastically alters the PL lifetime and essentially all the emission is quenched nonradiatively. The addition of d*t*Bubpy N^N ligand helps in improving the metal-ligand coupling but still most of the photons decay nonraditively as shown in Fig. 6c. Further addition of methoxy groups in **4a** and **4b** does not contribute to enhancing radiative decay. The PL decay characteristics are summerised in Table 1. The presence of iridium and the large observed stokes shifts, ranging from 7806–11606 cm^−1^, clearly indicate phosphorescence despite the short emission state lifetimes.

The markedly low quantum yields are accompanied by very short, nanosecond emission lifetimes. The structural commonality in these poorly emissive complexes is the presence of the 3-MeO groups on the C^N ligands. Similarly low luminescence had been previously reported for red-emitting *fac*-Ir(atpy)_3_, where atpy = 2-(5′-amino-4′-tolyl)pyridine (λ_max_ = 613 nm in DMSO, Φ_PL_ = 9.4 × 10^−3^)^58^. Thus, though substitution at this position red shifts the emission energy the most, electron donation into the Ir-C_C^N_ bond leads to rapid decay of the excited state. Generally, the τ_e_’s of **1b-4b** are nearly twofold longer than those of **1a-4a** due to the presence of the bulky *tert-*butyl groups.

**Figure 7:**
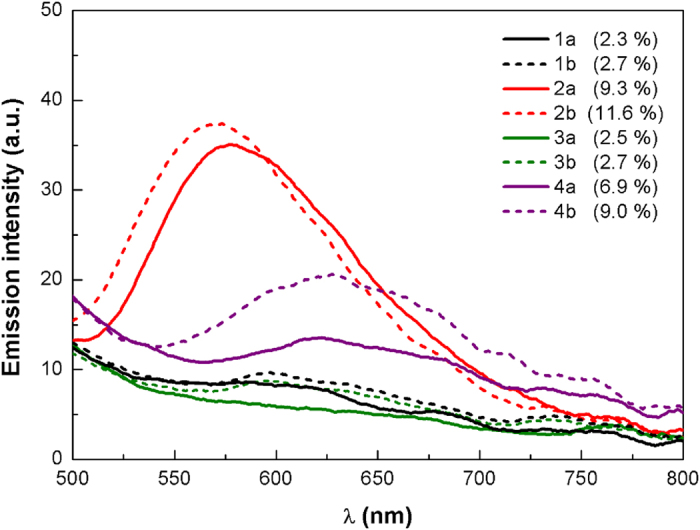
Photoluminescence spectra of complexes 1a-4b in thin solid films mixed with [BMIM^+^ :PF_6_^–^] in a molar ratio 4:1. The photoluminescence quantum yield (Φ_PL_) for the solid films are indicated in the legend (numbers in brackets).

The photoluminescence spectra in thin films for **1a-4b** were obtained by spin-coating an acetonitrile solution of each complex mixed with the ionic liquid (IL) 1-butyl-3-methylimidazolium hexafluorophosphate [BMIM^+^:PF_6_^−^], in a similar configuration to the light-emitting layer in LEECs (*vide infra*). The films were prepared on quartz substrates and recorded with λ_exc_ = 350 nm at room temperature (see Fig. 7).

In thin solid-films, the emission spectra are quite similar to the photoluminescence spectra obtained in acetonitrile solution, but showing much increased Φ_PL_. All the spectra have a maxima located between 570–640 nm, with the bluest emission corresponding to the **2a** and **2b** complexes at 575 nm and 580 nm, respectively, similar to those found in acetonitrile solution. These complexes present the highest PL intensities, exhibiting a Φ_PL_ close to 10% and 12%, respectively. However, the rest of the complexes show a red-shifted broad band centered around 640 nm that is significantly blue-shifted compared to their corresponding solution emission maxima, with PL intensities decreased drastically from **4a** and **4b** (Fig. 7).

### Theoretical calculations

In order to better understand the nature of the optoelectronic properties and the impact of methoxy substitution on the complexes, a combined density functional theory (DFT) and time-dependent DFT (TDDFT) study was undertaken^59^,^60^,^61^,^62^,^63^,^64^. All complexes were modeled using Gaussian 09^65^ using the following DFT protocol at the B3LYP^66^,^67^,^68^ level of theory with the SBKJC-DVZ^69^,^70^,^71^,^72^ basis set for iridium, 6–31 G* for heavy atoms directly coordinated to iridium and 3–21 G* for all other atoms^69^,^73^,^74^,^75^,^76^,^77^,^78^,^79^,^80^,^81^,^82^ in the presence of the solvent (ACN)^83^.

The geometry of the ground state structures was fully optimized without the imposition of symmetry restrictions. The geometry for each of the complexes is pseudo-octahedral. Selected structural parameters for **1a-4a** are summarized in Table 2. Bond lengths from the bonded ligand atoms to iridium are significantly longer than those observed for [Ir(ppy)_2_(bpy)]PF_6_: Ir–C_ppy_ = 2.013 Å, Ir–N_ppy_ = 2.045 Å, and Ir–N_bpy_ = 2.133 Å^84^. Ligand bite angles for **1a-4a** are similar to this archetype complex. Increasing the MeO-content on the C^N ligand results in a progressive contraction of the Ir-N_bpy_ bond. There is a modest elongation of the Ir-C^N bond in **4a**, most likely due to accommodation of the methoxy group at C_5_ of the phenyl ring.

**Figure 8:**
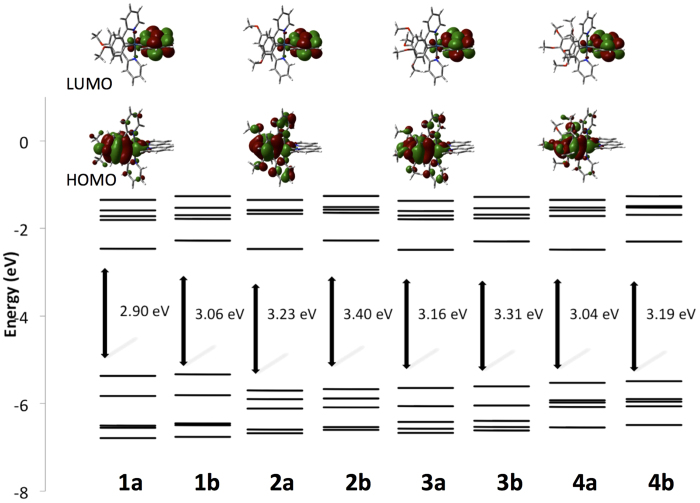
Calculated energy level scheme for the Kohn-Sham orbitals between HOMO-4 to LUMO+4 of 1a-4b, and the associated DFT calculated HOMO-LUMO energy gap (in eV) and electron density contour plots for 1a-4a (0.002 e bohr^−3^). The contour plots for **1b-4b** mirror those of **1a-4a**.

Figure 8 provides a comparison of the relative energies of the five highest energy occupied and five lowest energy unoccupied molecular orbitals (MOs) for **1a-4b** along with contour plots of HOMOs and LUMOs. As has been reported for analogous cationic systems^9^,^37^,^49^, the HOMO is located on both the aryl ring of the C^N ligands and the iridium atom. The 4-MeO group in **2a** and **2b** perturb this picture in that electron density is found also on the pyridyl moiety of the C^N ligand. The LUMO for each of the eight complexes is situated exclusively (electron density distribution >95%) on the diimine ligand.

The energies of the HOMOs are sensitive to the methoxy substitution pattern on the C^N ligands. For instance, the HOMO for **1a** resides at −5.37 eV whereas the HOMO for **2a** is significantly stabilized at −5.70 eV. When two MeO-groups are present as in **3a** there is only a slight destabilization to −5.64 eV compared to **2a**. A further destabilization is predicted for **4a** with the HOMO found at −5.53 eV. Replacement of bpy for d*t*Bubpy results in a net destabilization of the HOMO by only about 0.03 eV. Generally, the picture provided by the DFT computations is somewhat consistent with the CV data presented in Table S1 in that **2a** and **2b** are the most anodically shifted in the series of complexes. However, from a survey of the first oxidation potentials the DFT calculations underestimate the magnitude of the destabilization of the HOMO in **3a**, **3b**, **4a** and **4b**. The LUMO energies for **1a-4a** remain quite invariant at around −2.48 eV. In a similar fashion, the LUMO energies for **1b-4b** are destabilized relative to **1a-4a** owing to the electron-releasing *tert-*butyl substituents and are found around −2.29 eV. This trend is reproduced in the CVs though only a 0.08 V relative destabilization is observed experimentally. Similarly, the computations reproduce the principal features in the UV-Visible spectra for the complexes (cf. Figures S39-S42). The HOMO-LUMO energy gap is therefore smallest for **1a** at 2.90 eV and largest for **2b** at 3.40 eV.

**Figure 9:**
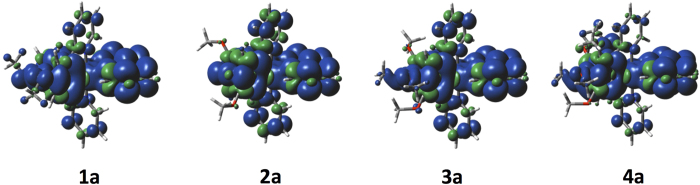
Calculated spin density contours of the *T*_1_ state for 1a, 2a, 3a and 4a (isocontour value of 0.0004 au).

The geometries of the triplet state were optimized using spin-unrestricted DFT calculations at the UB3LYP level. Notable deformations are modest contractions of the Ir-N_bpy_ and Ir-C_C^N_ bonds. Otherwise there is little perturbation of the geometry upon migrating onto the triplet manifold (Table 2). The spin densities for the *T*_1_ state for **1a-4a** are shown in Fig. 9; those for **1b-4b** possess similar topologies. The *T*_1_ state is describe predominantly by a LUMO →HOMO transition implying an emission resulting from an admixture of ^3^MLCT/^3^LLCT. This assignment is consistent with the TD-DFT results (Table S3) and the observed broad and unstructured emission at 298 K in ACN.

The emission energy was predicted using three different methodologies and these results are summarized in Table 3. The phosphorescence is estimated as the difference between the *T*_1_ and *S*_0_ states in their respective optimized geometries (E_0,0_), which is a good indicator of the E_0,0_ emission measured at 77 K. The emission predicted by TDDFT (E_TDDFT_) for the S0 → T1 monoexcitation are based on an optimized *S*_0_ geometry. The adiabatic electronic emission (E_AE_) is determined from the vertical energy difference between the *T*_1_ and *S*_0_ states at the optimized geometry of the *T*_1_ state. The E_AE_ calculations reproduce accurately the solution state emission observed at 298 K, with relative errors ranging from 3.1–15.4% though the calculations for **3a** (and **3b**) underestimate more significantly the impact of the electron-releasing methoxy groups on red shifting the emission.

### Device Characterization

Despite the poor solution and solid state PL performance and taking into account the presence of several candidate red emitters we investigated the electroluminescence performance of the iridium complexes by preparing simple two-layer light emitting electrochemical cells (LEECs). The devices were prepared by spin-coating a thin layer (80 nm) of poly(3,4-ethylenedioxythiophene):poly(styrenesulfonate) (PEDOT:PSS) on top of an indium tin oxide (ITO)-coated glass substrate, followed by the active layer (100 nm) consisting of a mixture of each complex with the ionic liquid (IL) 1-butyl-3-methylimidazolium hexafluorophosphate [BMIM^+^:PF_6_^–^] at a molar ratio of 4:1. The ionic liquid was added to reduce the turn-on time of the device, as higher mobilities for the ionic species are expected. Finally a 70 nm aluminium layer was evaporated as the top electrode. More details concerning the device preparation can be found in the Experimental section.

**Figure 10:**
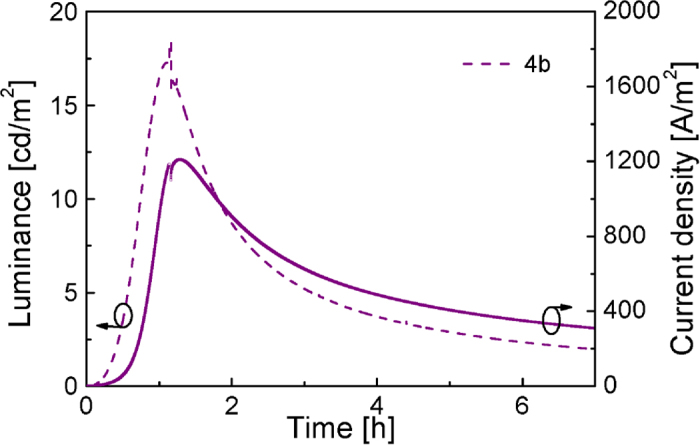
Electroluminescence performance for ITO/PEDOT:PSS/4b:IL(4:1)/Al device driven at a constant voltage of 4 V.

The device lifetime was measured by applying a constant voltage (4 V) and monitoring the current density and luminance versus time. Most of the devices didn´t show good performances, presenting very short lifetimes and efficiencies. The best result, obtained for molecule **4b**, is shown in Fig. 10.

As can be seen in Fig. 10, the luminance reaches a maximum value of 18 cd m^−2^ after one hour running, and decays to a half-maximum (L_1/2_) in 2 hours. The current peaks at 1206 A m^−2^ after 1.28 hours, and decreases in a similar trend to the luminance. As a consequence, low efficiencies were reached in these devices, leading to values lower than 1 cd A^−1^, an external quantum efficiency (EQE %) of 0.05% and CIE_(x,y)_ coordinates equal to (0.61, 0.38).

**Figure 11:**
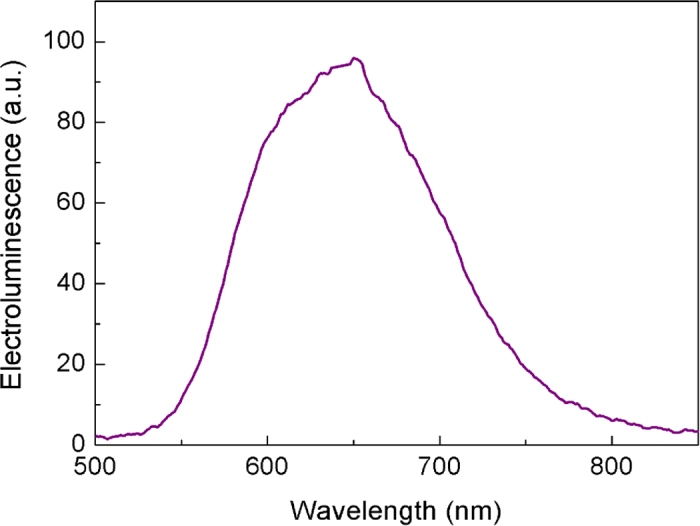
Electroluminescence spectra obtained for the LEEC device prepared with molecules 4b at 298 K.

Figure 11 shows the typical electroluminescence spectra for the LEEC device prepared with the molecule **4b**. Comparing with the previous data it resembles the PL spectra, which consist on a broad band centered at around 650 nm.

Device parameters for the other complexes in the series can be found in the supporting information (see Table S4). Compared to other recently reported LEECs, these performances are not very high^85^,^86^. In fact LEECs using complex **1a** had such low performance that no electroluminescence data could be obtained. Nevertheless, the complexes that are presented in this work are interesting emitters as they show a very red-shifted emission spectra with maxima around 650 nm, leading to very deep-red CIE coordinates.

## Conclusions

Herein, we reported eight new cationic iridium complexes bearing methoxy groups on the cyclometalating ligand. The goal of the study was to use mesomerically electron donating groups to promote a bathochromic shift in the emission and produce deep red emitters. Our measurements of solutions of these materials show that through incorporation of a methoxy group *para* to the Ir-C bond in the complex deep red to near infrared emission could be obtained in the complexes, however at dramatically reduced photoluminescence quantum yields and emission lifetimes. Experimental results are consistent with the DFT calculations. PL intensity increased somewhat in the thin film and LEEC devices were fabricated. Though these LEEC devices were also found to emit in the deep red they were poorly stable and not bright. From this study we can conclude that using electron donating groups on the cyclometallating ligands in cationic iridium complexes is not a good design strategy for producing bright and stable red emitters and rather highly conjugative C^N ligands should be employed.

## Experimental Section

### General Synthetic Procedures

Commercial chemicals were used as supplied. All reactions were performed using standard Schlenk techniques under inert (N_2_) atmosphere with freshly distilled anhydrous solvents obtained from a Pure Solv^TM^ solvent purification system from Innovative Technologies except where specificallsy mentioned. Flash column chromatography was performed using silica gel (Silia-P from Silicycle, 60 Å, 40–63 *μ*m). Analytical thin layer chromatography (TLC) was performed with silica plates with aluminum backings (250 *μ*m with indicator F-254). Compounds were visualized under UV light. ^1^H and ^13^C NMR spectra were recorded on a Brucker Avance spectrometer at 400 MHz and 100 MHz, respectively. The following abbreviations have been used for multiplicity assignments: “s” for singlet, “d” for doublet, “t” for triplet, “m” for multiplet and “br” for broad. Deuterated chloroform (CDCl_3_) and deuterated actonitrile (CD_3_CN) were used as the solvent of record. Melting points (Mp’s) were recorded using open-ended capillaries on a Meltemp melting point apparatus and are uncorrected. High resolution mass spectra were recorded on a quadrupole time-of-flight (ESI-Q-TOF), model Maxis from Bruker in positive electrospray ionization mode and spectra were recorded using sodium formate solution as calibrantat at the Université de Sherbrooke. *m*-Methoxybenzeneboronic acid, *p*-Methoxybenzeneboronic acid, dimethoxybenzeneboronic acid and 3,4,5-Trimethoxybenzeneboronic acid were prepared in 75, 78, 36 and 65%, respectively from the corresponding bromomethoxybenzenes following the reported literature procedure^87^. The corresponding iridium(III) dimers, [(C^N)_2_Ir(Cl)]_2_ were prepared according to the procedure described by Nonoyama^42^ and isolated by filtration and used without further purification or characterization.

### General Procedure for the Synthesis of cyclometallating (C^N) ligands

2-Bromo-5-methylp-yridine (1.0 equiv.), K_2_CO_3_ (3.0 equiv.) and arylboronic acid (1.25 equiv.) were dissolved in degassed dioxane/H_2_O (5:1 v/v) in order to obtain a 0.05 M concentration in 2-bromo-5-methylpyridine. Pd(PPh_3_)_4_ (0.008 g, 0.007 mmol, 5 mol%) was then added and the solution was repeatedly degassed. The reaction mixture was heated at reflux temperature for 19 h. Upon cooling, the solution was quenched with equal volume of H_2_O and diluted with equal volume of DCM. The organic layer was separated and dried over anhydrous MgSO_4._ Removal of solvent gave a dark slurry, which was purified by column chromatography with silica gel (EtOAc/hexane, 1:9 to 1:1)

**2-(3’-Methoxyphenyl)-5-methylpyridine (3-MeOppy).** 0.026 g was obtained as yellow oil-like liquid. **Yield:** 95%. *R*_*f*_: 0.23 (10% EtOAc/hexanes on silica). ^**1**^**H NMR (400 MHz, CDCl**_**3**_**) δ (ppm):** 8.51 (dd, *J* = 0.70, 1.85 Hz, 1H), 7.61 (d, *J* = 8.06 Hz, 1H), 7.54 (m, 2H), 7.51 (ddd, *J* = 0.87, 1.46, 7.68, 1H), 6.94 (t, *J* = 8.17 Hz, 1H), 3.88 (s, 3H), 2.36 (s, 3H). The characterization matches that previously reported^88^.

**2-(4’-Methoxyphenyl)-5-methylpyridine (4-MeOppy)**: 0.675 g was obtained as a white solid. **Yield:** 95%. *R*_*f*_: 0.25 (10% EtOAc/hexanes on silica). ^**1**^**H NMR (400 MHz, CDCl**_**3**_**) δ (ppm):** 8.48 (s, 1H), 7.92 (m, 2H), 7.54 (dt, *J* = 4.95, 8.17 Hz, 2H), 6.99 (m, 2H), 3.86 (s, 3H), 2.35 (s, 3H). The characterization matches that previously reported^89^,^90^.

**2-(3’,4’-Dimethoxyphenyl)-5-methylpyridine (3,4-dMeO):** 0.602 g was obtained as a brownish damp solid. **Yield:** 99%. *R*_*f*_: 0.20 (10% EtOAc/hexanes on silica). ^**1**^**H NMR (400 MHz, CDCl**_**3**_**) δ (ppm):** 8.48 (s, 1H), 7.61 (m, 2H), 7.53 (dd, *J* = 2.05, 8.09 Hz, 1H), 7.47 (dd, *J* = 2.03, 8.35, 1H), 6.94 (d, *J* = 8.38, 1H), 3.99 (s, 3H), 3.93 (s, 3H), 2.26 (s, 3H). ^**13**^**C NMR (100 MHz, CDCl**_**3**_**) δ (ppm):** 154.7, 150.0, 149.9, 149.4, 137.6, 132.6, 131.3, 119.8, 119.3, 111.2, 109.9, 56.2, 18.4. **HR-MS (EI, 70 eV): [M + Na]**^**+**^ **Calculated:** (C_14_H_15_NO_2_Na) 252.0995; **Found:** 252.1003.

**2-(3’,4’,5’-Trimethoxyphenyl)-5-methylpyridine (3,4,5-tMeO):** 0.919 g was obtained as a yellow oil-like liquid. **Yield:** 99%. *R*_*f*_: 0.15 (10% EtOAc/hexanes on silica). ^**1**^**H NMR (400 MHz, CDCl**_**3**_**) δ (ppm):** 8.49 (dd, *J* = 0.73, 1.30, 1H), 7.55 (m, 2H), 7.20 (s, 2H), 3.95 (s, 6H), 3.89 (s, 3H), 2.37 (s, 3H). ^**13**^**C NMR (100 MHz, CDCl**_**3**_**) δ (ppm):** 154.6, 153.7, 150.0, 138.9, 137.6, 135.3, 131.8, 120.1, 104.0, 93.2, 61.1, 56.4, 18.4. **HR-MS (**ES-Q-TOF**): [M + Na]**^**+**^ **Calculated:** (C_15_H_17_NO_3_Na) 282.1101; **Found:** 282.1110.

### General procedure for the synthesis of [(C^N)_2_Ir(N^N)]PF_6_ complexes

Iridium dimer (0.07 mmol, equiv.) and N^N ligand (2,2’-bipyridine, bpy or 4,4’-di-*tert*-butyl-2,2’-bipyridine, d*t*bubpy) (0.16 mmol, 2.25 equiv.) were solubilized with 12 mL of DCM/MeOH (50:50 v/v). The mixture was degassed repeatedly and placed under N_2_ and heated to 55 °C for 19 h. Over the course of the reaction the mixture darkened in color. The solution was cooled to RT and the solvent removed under reduced pressure. The crude solid was re-dissolved in a minimum amount of MeOH and added slowly to an aqueous solution of NH_4_PF_6_ (10 mL, 6.13 mmol, 1 g/10 mL) under gentle stirring. The first drop caused the precipitation of a brownish yellow-to-red colored solid. The solid suspension was conserved at 0 °C for 2 h and then filtered through a Buckner funnel and the resulting solid was washed with water and Et_2_O. The residue was dried under *vacuo* to obtain the desired colored complex complex. The complex was then recrystallized in dichloromethane / di*iso*propylether (50:50 v/v) by slow evaporation.

**Iridium (III) bis [2-(3’-methoxyphenyl)-5-methylpyridinato-N,C**^**2’**^**]-*****N,N’*****-(bipyridine) hexafluorophosphate: [(3-MeOppy)**_**2**_**Ir(bpy)](PF**_**6**_**) 1a**: Red crystals (0.082 g). **Yield**: 94%. **Mp:** 310–311 °C. ^**1**^**H NMR (400 MHz, CD**_**3**_**CN) δ (ppm):** 8.53 (d, *J* = 8.17 Hz, 2H), 8.13 (t, *J* = 8.05 Hz, 2H), 8.02 (d, *J* = 5.43 Hz, 2H), 7.97 (d, *J* = 8.34 Hz, 2H), 7.69 (d, *J* = 8.32 Hz, 2H), 7.51 (dt, *J* = 1.17, 7.61 Hz, 2H), 7.38 (m, 4H), 6.62 (dd, *J* = 2.76, 8.29 Hz, 2H), 6.15 (d, *J* = 8.28, 2H), 3.79 (s, 6H), 2.12 (s, 6H). ^**13**^**C NMR (100 MHz, CDCl**_**3**_**) δ (ppm):** 165.2, 156.4, 156.0, 150.5, 148.2, 144.1, 139.9, 139.3, 139.2, 133.9, 132.0, 128.3, 125.4, 119.6, 117.3, 110.2, 55.6, 18.5**. HR-MS (**ES-Q-TOF**): [M-PF**_**6**_]^**+**^ **Calculated:** (C_36_H_32_IrN_4_O_2_) 745.2151; **Found:** 745.2160.

**Iridium (III) bis [2-(3’-methoxyphenyl)-5-methylpyridinato-N,C**^**2’**^**]-*****N,N’*****-(di-*****tert*****-bipyridine) hexafluorophosphate: [(3-MeO-ppy)**_**2**_**Ir(d*****t*****bubpy)](PF**_**6**_**) 1b**: Yellow powder (0.141 g). **Yield**: 88%. **Mp:** 209–210 °C. ^**1**^**H NMR (400 MHz, CD**_**3**_**CN) δ (ppm):** 8.49 (d, *J* = 1.83 Hz, 2H), 7.97 (d, *J* = 8.43 Hz, 2H), 7.89 (d, *J* = 5.87 Hz, 2H), 7.69 (dd, *J* = 1.10, 8.43 Hz, 2H), 7.51 (dd, *J* = 2.20, 5.87 Hz, 2H), 7.36 (m, 4H), 6.62 (dd, *J* = 2.57, 8.43 Hz, 2H), 6.13 (d, *J* = 8.07, 2H), 3.78 (s, 6H), 2.12 (s, 6H), 1.43 (s, 18H). ^**13**^**C NMR (100 MHz, CDCl**_**3**_**) δ (ppm):** 165.2, 164.0, 156.2, 156.1, 150.0, 148.5, 144.3, 139.8, 139.1, 133.8, 131.9, 125.5, 121.8, 119.4, 117.2, 110.1, 105.9, 55.6, 36.0, 30.9, 30.5, 18.5**. HR-MS (**ES-Q-TOF**): [M-PF**_**6**_]^**+**^ **Calculated:** (C_44_H_48_IrN_4_O_2_) 857.3404; **Found:** 857.3401.

**Iridium (III) bis [2-(4’-methoxyphenyl)-5-methylpyridinato-N,C**^**2’**^**]-*****N,N’*****-(bipyridine) hexafluorophosphate: [(4-MeOppy)**_**2**_**Ir(bpy)](PF**_**6**_**) 2a**: Yellow powder (0.134 g). **Yield**: 87%. **Mp:** 342 °C. ^**1**^**H NMR (400 MHz, CD**_**3**_**CN) δ (ppm):** 8.54 (d, *J* = 8.18 Hz, 2H), 8.14 (dt, *J* = 1.60, 8.00 Hz, 2H), 8.05 (d, *J* = 5.86 Hz, 2H), 7.84 (d, *J* = 8.39 Hz, 2H), 7.71 (d, *J* = 8.62 Hz, 2H), 7.64 (dd, *J* = 1.42, 8.36, 2H), 7.52 (dt, *J* = 1.16, 7.57, 2H), 7.33 (s, 2H), 6.64 (dd, *J* = 2.57, 8.60 Hz, 2H), 5.72 (d, *J* = 2.56 Hz, 2H), 3.59 (s, 6H), 2.09 (s, 6H). ^**13**^**C NMR (100 MHz, CDCl**_**3**_**) δ (ppm):** 165.1, 161.0, 155.9, 152.0, 150.4, 147.7, 140.0, 139.2, 137.0, 132.3, 128.2, 126.1, 125.6, 118.7, 117.6, 107.4, 55.0, 18.5**. HR-MS (**ES-Q-TOF**): [M-PF**_**6**_]^** + **^**Calculated:** (C_36_H_32_IrN_4_O_2_) 745.2151; **Found:** 745.2147. This photophysical characterization of this complex has been previously reported (λ_em_ = 593 nm, Φ_PL_ = 2.19%, τ_e_ = 217 ns) but not the compound characterization^41^.

**Iridium (III) bis [2-(4’-methoxyphenyl)-5-methylpyridinato-N,C**^**2’**^**]-*****N,N’*****-(di-*****tert*****-bipyridine) hexafluorophosphate: [(4-MeOppy)**_**2**_**Ir(d*****t*****bubpy)](PF**_**6**_**) 2b**: Yellow powder (0.134 g). **Yield**: 84%. **Mp:** 236–238 °C. ^**1**^**H NMR (400 MHz, CD**_**3**_**CN) δ (ppm):** 8.50 (d, *J* = 1.78 Hz, 2H), 7.92 (d, *J* = 5.86 Hz, 2H), 7.84 (d, *J* = 8.38 Hz, 2H), 7.70 (d, *J* = 8.61 Hz, 2H), 7.65 (m, 2H), 7.53 (dd, *J* = 1.88, 5.85 Hz, 2H), 7.28 (s, 2H), 6.63 (dd, *J* = 2.56, 8.59, 2H), 5.70 (d, *J* = 2.53 Hz, 2H), 3.59 (s, 6H), 2.10 (s, 6H), 1.44 (s, 18H). ^**13**^**C NMR (100 MHz, CDCl**_**3**_**) δ (ppm):** 165.1, 164.1, 160.9, 155.9, 152.6, 149.9, 148.0, 139.1, 137.2, 132.4, 126.0, 125.5, 122.0, 118.6, 117.3, 107.4, 105.9, 55.0, 36.0, 18.5**. HR-MS (**ES-Q-TOF**): [M-PF**_**6**_]^** + **^**Calculated:** (C_44_H_48_IrN_4_O_2_) 857.3404; **Found:** 857.3403. This photophysical characterization of this complex has been previously reported (λ_em_ = 576 nm, Φ_PL_ = 5.87%, τ_e_ = 401 ns) but not the compound characterization^41^.

**Iridium (III) bis [2-(3’,4’-dimethoxyphenyl)-5-methylpyridinato-N,C**^**2’**^**]-*****N,N’*****-(bipyridine) hexafluorophosphate: [(3,4-dMeOppy)**_**2**_**Ir(bpy)](PF**_**6**_**) 3a**: Brownish yellow powder (0.132 g). **Yield**: 95%. **Mp:** 234–236 °C. ^**1**^**H NMR (400 MHz, CD**_**3**_**CN) δ (ppm):** 8.52 (d, *J* = 8.16 Hz, 2H), 8.13 (dt, *J* = 1.54, 8.02 Hz, 2H), 8.04 (d, *J* = 5.35 Hz, 2H), 7.84 (d, *J* = 8.39 Hz, 2H), 7.63 (dd, *J* = 1.32, 8.41 Hz, 2H), 7.53 (dt, *J* = 1.16, 7.57, 2H), 7.35 (s, 4H), 5.74 (s, 2H), 3.82 (s, 6H), 3.45 (s, 6H), 2.10 (s, 6H). ^**13**^**C NMR (100 MHz, CD**_**3**_**CN) δ (ppm):** 165.5, 156.3, 151.3, 151.1, 148.7, 145.8, 142.8, 139.2, 135.7, 132.4, 128.4, 124.8, 117.6, 113.6, 109.4, 100.6, 56.0, 54.9, 17.2**. HR-MS (**ES-Q-TOF**): [M-PF**_**6**_]^**+**^ **Calculated:** (C_38_H_36_IrN_4_O_4_) 805.2362; **Found:** 805.2361.

**Iridium (III) bis [2-(3’,4’-dimethoxyphenyl)-5-methylpyridinato-N,C**^**2’**^**]-*****N,N’*****-(di-*****tert*****-bipyridine) hexafluorophosphate: [(3,4-dMeOppy)**_**2**_**Ir(d*****t*****bubpy)](PF**_**6**_**) 3b**: Brownish yellow powder (0.158 g). **Yield**: 98%. **Mp:** 318 °C. ^**1**^**H NMR (400 MHz, CD**_**3**_**CN) δ (ppm):** 8.50 (d, *J* = 1.65 Hz, 2H), 7.92 (d, *J* = 5.84 Hz, 2H), 7.84 (d, *J* = 8.40 Hz, 2H), 7.63 (dd, *J* = 1.36, 8.43 Hz, 2H), 7.53 (dd, *J* = 1.88, 5.85, 2H), 7.34 (s, 2H), 7.32 (s, 2H), 5.73 (s, 2H), 3.82 (s, 6H), 3.44 (s, 6H), 2.11 (s, 6H), 1.44 (s, 18H). ^**13**^**C NMR (100 MHz, CDCl**_**3**_**) δ (ppm):** 165.8, 164.0, 156.0, 151.1, 150.0, 148.1, 145.5, 143.2, 139.0, 135.2, 132.0, 125.6, 121.7, 118.6, 113.1, 108.4, 105.9, 56.3, 55.5, 36.0, 30.8, 30.5, 18.4**. HR-MS (**ES-Q-TOF**): [M-PF**_**6**_]^**+**^ **Calculated:** (C_46_H_52_IrN_4_O_4_) 917.3615; **Found:** 917.3612.

**Iridium (III) bis [2-(3’,4’,5’-trimethoxyphenyl)-5-methylpyridinato-N,C**^**2’**^**]-*****N,N’*****-(bipyridine) hexafluorophosphate: [(3,4,5-tMeOppy)**_**2**_**Ir(bpy)](PF**_**6**_**) 4a**: Red powder (0.107 g). **Yield**: 98%. **Mp:** 203 °C. ^**1**^**H NMR (400 MHz, CD**_**3**_**CN) δ (ppm):** 8.49 (d, *J* = 8.14 Hz, 2H), 8.13 (t, *J* = 7.34 Hz, 2H), 8.05 (d, *J* = 5.16 Hz, 2H), 7.84 (d, *J* = 8.39 Hz, 2H), 7.52 (dd, *J* = 6.7712.22 Hz, 4H), 7.30 (s, 2H), 7.14 (s, 2H), 3.90 (s, 6H), 3.74 (s, 6H), 2.87 (s, 6H), 2.01 (s, 6H). ^**13**^**C NMR (100 MHz, CDCl**_**3**_**) δ (ppm):** 165.3, 159.1, 156.1, 150.4, 148.5, 144.2, 140.1, 140.0, 138.2, 131.7, 130.5, 128.4, 125.6, 118.5, 105.0, 61.0, 60.8, 56.5, 18.5**. HR-MS (**ES-Q-TOF**): [M-PF**_**6**_]^**+**^ **Calculated:** (C_40_H_40_IrN_4_O_6_) 865.2574; **Found:** 865.2572.

**Iridium (III) bis [2-(3’,4’,5’-trimethoxyphenyl)-5-methylpyridinato-N,C**^**2’**^**]-*****N,N’*****-(di-*****tert*****-bipyri-dine) hexafluorophosphate: [(3,4,5-tMeOppy)**_**2**_**Ir(d*****t*****bubpy)](PF**_**6**_**) 4b**: Brownish yellow powder (0.107 g). **Yield**: 75%. **Mp:** 186–188 °C. ^**1**^**H NMR (400 MHz, CD**_**3**_**CN) δ (ppm):** 8.46 (s, 2H), 7.92 (d, *J* = 5.79 Hz, 2H), 7.83 (d, *J* = 8.36 Hz, 2H), 7.53 (t, *J* = 6.16 Hz, 4H), 7.04 (s, 2H), 3.90 (s, 6H), 3.74 (s, 6H), 2.87 (s, 6H), 2.01 (s, 6H), 1.42 (s, 18H). ^**13**^**C NMR (100 MHz, CDCl**_**3**_**) δ (ppm):** 206.6, 165.4, 164.4, 159.1, 156.0, 150.2, 149.8, 148.6, 144.1, 140.3, 138.1, 131.6, 131.0, 125.8, 121.8, 118.6, 61.0, 60.8, 56.5, 36.0, 30.5 18.5**. HR-MS (**ES-Q-TOF**): [M-PF**_**6**_]^**+**^ **Calculated:** (C_48_H_56_IrN_4_O_6_) 977.3827; **Found:** 977.3829.

### Photophysical measurements

All samples were prepared in HPLC grade acetonitrile (ACN) with varying concentrations on the order of *μ*M. Absorption spectra were recorded at RT using a Shimadzu UV-1800 double beam spectrophotometer. Molar absorptivity determination was verified by linear least-squares fit of values obtained from at least three independent solutions at varying concentrations with absorbance ranging from 6.88 × 10^−1^ to 3.19 × 10^2^ μM.

The sample solutions for the emission spectra were prepared in N_2_-degassed dry ACN. Emission spectra were recorded at room temperature using a Cary Eclipse 300 fluorimeter. The samples were excited at the absorption maxima of the dominant low-energy ^1^MLLCT band as indicated in Table 1. Excited state lifetimes were measured with an Edinburgh Instruments Mini Tau lifetime fluorimeter with an EPL 405 laser (exciting at 405 nm). Melting points were measured with a BI Barnsted Electrothermal 9100 apparatus and were quoted referencing the decomposition temperature. Emission quantum yields were determined using the optically dilute method^56^,^91^. A stock solution with absorbance of ca. 0.5 was prepared and then four dilutions were prepared with dilution factors of 40, 20, 13.3 and 10 to obtain solutions with absorbances of ca. 0.013 0.025, 0.038 and 0.05, respectively. The Beer-Lambert law was found to be linear at the concentrations of the solutions. The emission spectra were then measured after the solutions were rigorously degassed with solvent-saturated nitrogen gas (N_2_) for 20 minutes prior to spectrum acquisition using septa-sealed quartz cells from Starna. For each sample, linearity between absorption and emission intensity was verified through linear regression analysis and additional measurements were acquired until the Pearson regression factor (R^2^) for the linear fit of the data set surpassed 0.9. Individual relative quantum yield values were calculated for each solution and the values reported represent the slope value. The equation Φ_s_ = Φ_r_(*A_r_/A_s_*)(*I_s_/I_r_*)(*ns*/*n*_r_)^2^ was used to calculate the relative quantum yield of each of the sample, where Φ_r_ is the absolute quantum yield of the reference, *n* is the refractive index of the solvent, *A* is the absorbance at the excitation wavelength, and *I* is the integrated area under the corrected emission curve. The subscripts s and r refer to the sample and reference, respectively. A solution of [Ru(bpy)_3_](PF_6_)_2_ in ACN (Φ_r_ = 0.095) was used as the external reference^55^.

### Electrochemistry measurements

Cyclic voltammetry (CV) measurements were performed on an Electrochemical Analyzer potentiostat model 600D from CH Instruments. Solutions for cyclic voltammetry were prepared in ACN and degassed with ACN-saturated nitrogen bubbling for about 10 min prior to scanning. Tetra(*n*-butyl)ammoniumhexafluorophosphate (TBAPF_6_; ca. 0.1 M in ACN) was used as the supporting electrolyte. A non-aqueous Ag/Ag^+^ electrode (silver wire in a solution of 0.1 M AgNO_3_ in ACN) was used as the pseudoreference electrode; a glassy-carbon electrode was used for the working electrode and a Pt electrode was used as the counter electrode. The redox potentials are reported relative to a normal hydrogen electrode (NHE) electrode with a ferrocenium/ferrocene (Fc^+^ /Fc) redox couple as an internal reference (0.38 V vs SCE)^43^.

### Density Functional Theory (DFT) Calculations

All calculations were performed with the Gaussian 09^92^ suite. The level of theory for all DFT^61^,^93^,^94^,^95^ and TD-DFT^62^,^63^,^64^ calculations was B3LYP; excited-state triplet geometries were calculated using the unrestricted B3LYP method (UB3LYP)^67^,^68^,^96^. The 6-31G* basis set^97^ was used for C, H and N directly linked to Iridium while the other C, H, N and F atoms where undertaken with 3-21G* basis set^69^,^73^,^74^,^75^,^76^,^77^, and the VDZ (valence double ζ) with SBKJC effective core potential basis set^69^,^70^,^71^,^72^ was used for Iridium. The predicted phosphorescence wavelengths were obtained by energy difference between the Triplet and Singlet states at their respective optimized geometries^41^,^49^. The energy, oscillator strength and related MO contributions for the 100 lowest singlet-singlet and 5 lowest singlet-triplet excitations were obtained from the TD-DFT/Singlets and the TD-DFT/Triplets output files, respectively. The calculated absorption spectra were visualized with GaussSum 2.1 (fwhm: 1000 cm^−1^)^98^.

### Device Fabrication

An aqueous dispersion of Poly(3,4-ethylenedioxythiophene): poly-styrenesulfonate (PEDOT:PSS) was purchased from Hereaus and used as received. The ionicliquid 1-butyl-3-methyl-imidazolium hexafluorophosphate [BMIM^+^][PF^−6^] and the solvents used were obtained from Aldrich. Photolithographically patterned ITO covered glass substrates were purchased from Naranjo-Substrates (www.naranjosubstrates.com) and extensively cleaned before using by ultrasonic treatment in water-soap, water, and 2-propanol baths. After drying, the substrates were placed in a UV-ozone cleaner (Jelight 42-220) for 20 min.

Devices were prepared on the cleaned ITO substrates by depositing an 80 nm layer of PEDOT:PSS and annealing it at 150 °C during 15 minutes. On top of this layer the active film was deposited from an acetonitrile solution of the complex mixed with the ionic liquid in a molar ratio 4 to 1. All the layers were prepared by spin-coating the organic solutions. A concentration of 20 mg mL^−1^ of iTMC at 1000 rpm for 30 seconds leads to 100 nm thicknes. The thickness of the films was determined using an Ambios XP1 profilometer. Then, the samples were transferred to an inert atmosphere glovebox (<0.1 ppm O_2_ and H_2_O, MBraun). Finally, aluminum metal electrodes (70 nm) were thermally evaporated using a shadow mask under a vacuum (<1 ×  10^−6^ mbar) using an Edwards Auto500 evaporator integrated into an inert atmosphere glovebox. Lifetime data were obtained by applying pulsed currents and monitoring the voltage and simultaneously the luminance by a True Colour Sensor MAZeT (MTCSICT Sensor) using a Lifetime Test System designed by BoTEST (Botest OLT OLED Lifetime-Test System). Electroluminescence spectra were recorded using an Avantis fiberoptics photospectrometer. The devices were not encapsulated and were characterized inside the glovebox.

## Additional Information

**How to cite this article**: Hasan, K. *et al.* Tuning the Emission of Cationic Iridium (III) Complexes Towards the Red Through Methoxy Substitution of the Cyclometalating Ligand. *Sci. Rep.* **5**, 12325; doi: 10.1038/srep12325 (2015).

## Supplementary Material

Supplementary Information

## Figures and Tables

**Table 1:** Relevant photophysical data for complex 1a-4b.

Complex	λ_em_ (nm)^a^	Ф_PL_ (%)^b^	τ_1_ (ns)^a^	Α_1_^c^	τ_2_ (ns)^a^	Α_2_^c^
**1a**	710	0.2	4.72	1	–	–
**1b**	680	0.3	10.2	0.84	174	0.16
**2a**	618	5.7	194	0.97	1505	0.03
**2b**	595	15.4	388	0.96	2411	0.04
**3a**	730	0.3	1.6	0.95	29.3	0.05
**3b**	700	0.1	3.2	0.82	100	0.18
**4a**	720	0.2	1.66	0.91	23.3	0.09
**4b**	685	0.2	3.2	0.78	70	0.22

^a^Measured in deaerated ACN at 298 K at excitation wavelength 390 nm.

^b^Using [Ru(bpy)_3_](PF_6_)_2_ as the standard (Ф_PL_ = 9.5% in deaerated ACN at 298 K).

^c^A_1_ and A_2_ are the pre-exponential factors for the phosphorescence lifetime.

**Table 2:** Selected average structural parameters for 1a, 2a, 1b, and 2b^a^.

Complexes	1a		2a		3a		4a	
	S_0_	T_1_	S_0_	T_1_	S_0_	T_1_	S_0_	T_1_
Ir-N_bpy_	2.187	2.179	2.186	2.175	2.184	2.175	2.178	2.169
Ir-N_C^N_	2.077	2.076	2.078	2.075	2.078	2.076	2.073	2.072
Ir-C_C^N_	2.030	2.002	2.028	2.008	2.028	2.003	2.039	2.011
N_bpy_-Ir-N_bpy_	75.5	76.0	75.6	76.1	75.7	76.1	75.9	76.4
N_C^N_-Ir-N_C^N_	80.2	80.9	80.3	81.2	80.2	81.0	79.9	80.3

^a^Bond lengths in Å and bond angles in°.

**Table 3:** Predicted Emission Energies.

	Theoretical^a^			λ_em_ (298 K) /nm	Error^d^*/%*
	E_TDDFT_/nm	E_0,0_/nm	E_AE_/nm		
**1a**	550.7	593.0	688.4	710	3.1
**2a**	483.8	507.9	566.0	618	8.4
**3a**	499.6	535.5	618.0	730	15.4
**4a**	524.1	574.0	676.6	720	6.0

^*a*^E_TDDFT_ = energy of S_0_ →T_1_ transition obtained by TDDFT at the *S*_0_ optimized geometry; E_0,0_ = E(*T*_1_)-E(*S*_0_) at their respective optimized geometries obtained by DFT; E_AE_ = E(*T*_1_)-E(*S*_0_) at the *T*_1_ optimized geometries (adiabatic electronic emission) obtained by DFT. See experimental section for computational details. All values obtained are in the presence of ACN solvent; ^*b*^Highest energy 77 K emission band reported and highest intensity 298 K emission band reported; ^*c*^Error = |λ_em_(77 K)-E_0,0_/λ_em_(77 K)| in eV; ^*d*^Error = |λ_em_(298 K)-E_AE_/λ_em_(298 K)| in eV.
