# Supplementary material for: Tuning the Emission of Cationic Iridium (III) Complexes Towards the Red Through Methoxy Substitution of the Cyclometalating Ligand
Source: Sci Rep. 2015 Jul 16;5:12325. doi: 10.1038/srep12325 (PMC4648437; doi:10.1038/srep12325)
Supplement: Supplementary Information [file srep12325-s1.pdf]

Tuning the Emission of Cationic Iridium (III) Complexes Towards the Red Through Methoxy Substituion of the Cyclometalating Ligand

Kamrul Hasan,[a] Ashu K. Bansal,[b] Ifor D.W. Samuel,[b] Cristina Roldán-Carmona,[c,d] Henk Bolink[c] and Eli Zysman-Colman[e]**

[a] *Département de Chimie, Université de Sherbrooke, 2500 Boul. de l'Université, Sherbrooke, QC, Canada, J1K 2R1*

[b] Organic Semiconductor Centre, SUPA School of Physics and Astronomy, University of St. Andrews, North Haugh, St. Andrews, Fife, UK, KY16 9SS

[c] *Instituto de Ciencia Molecular, Universidad de Valencia, C/ Catedrático J. Beltrán 2, 46980 Paterna (Valencia), Spain. henk.bolink@uv.es*

[d] *Department of Physical Chemistry and Applied Thermodynamics, Campus Rabanales, Ed. C3, University of Cordoba, 14014, Spain*

[e] Organic Semiconductor Centre, EaStCHEM School of Chemistry, University of St Andrews, St Andrews, Fife, UK, KY16 9ST, Fax: +44-1334 463808; Tel: +44-1334 463826; E-mail: eli.zysman-colman@st-andrews.ac.uk; URL: http://www.zysman-colman.com

SUPPORTING INFORMATION

Table of contents:

	Pages
^{1}H and ^{13}C NMR spectrum of individual compounds and complexes	S2-S23
Synthesis of ligands	S24
Tables of electrochemical and UV-Visible absorption data	S24-S25
UV-vis and emission spectra of individual complexes	S26-S29
Cyclic voltammograms of individual complexes	S30-S33
TDDFT UV-Vis absorption spectra predictions	S34-S37
Performance of LEECs fabricated with **1a** – **4b** complexes	S38
References	S38

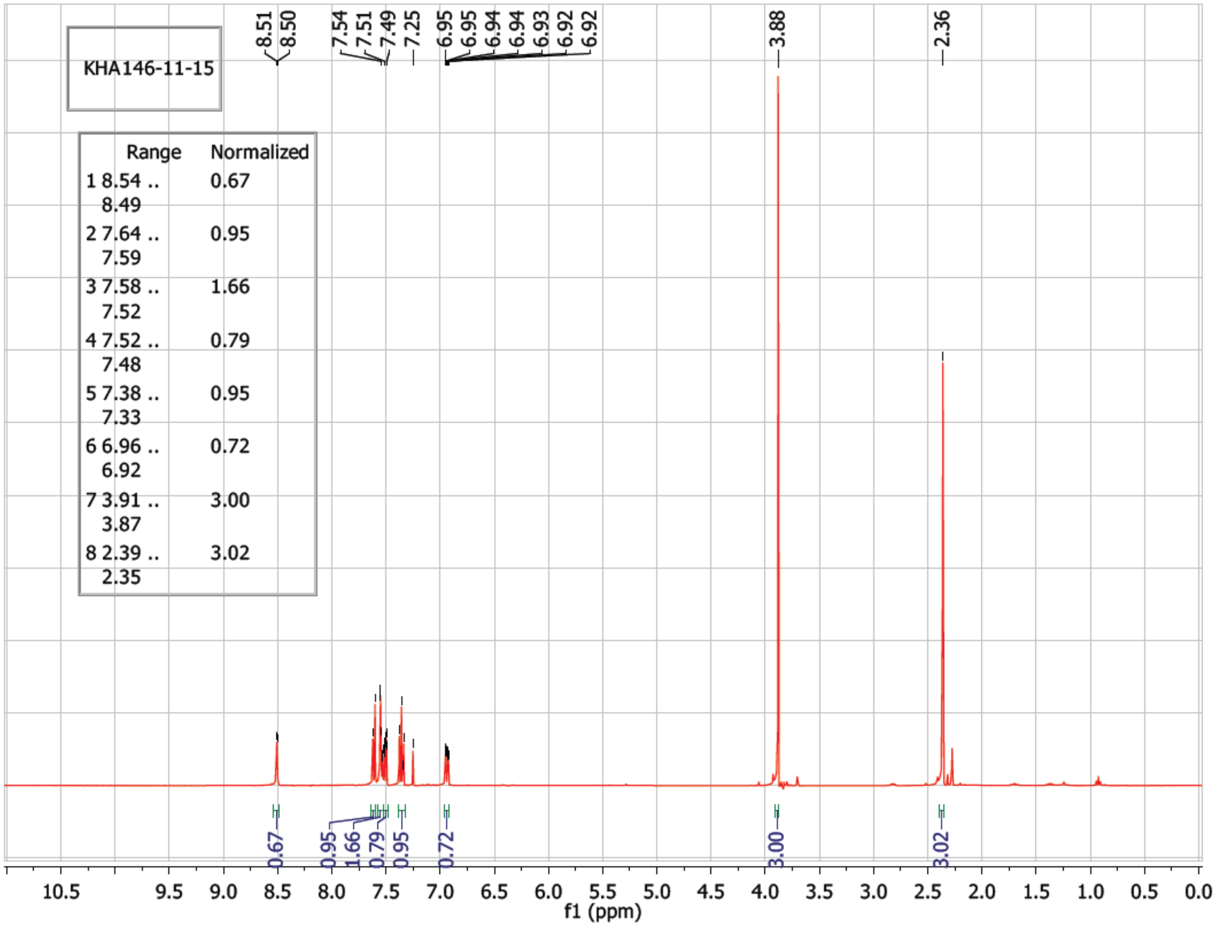

Figure **S1**. ^{1}H NMR spectrum of **2-(3'-Methoxyphenyl)-5-methylpyridine (3-MeOppy)** in $CDCl_3$

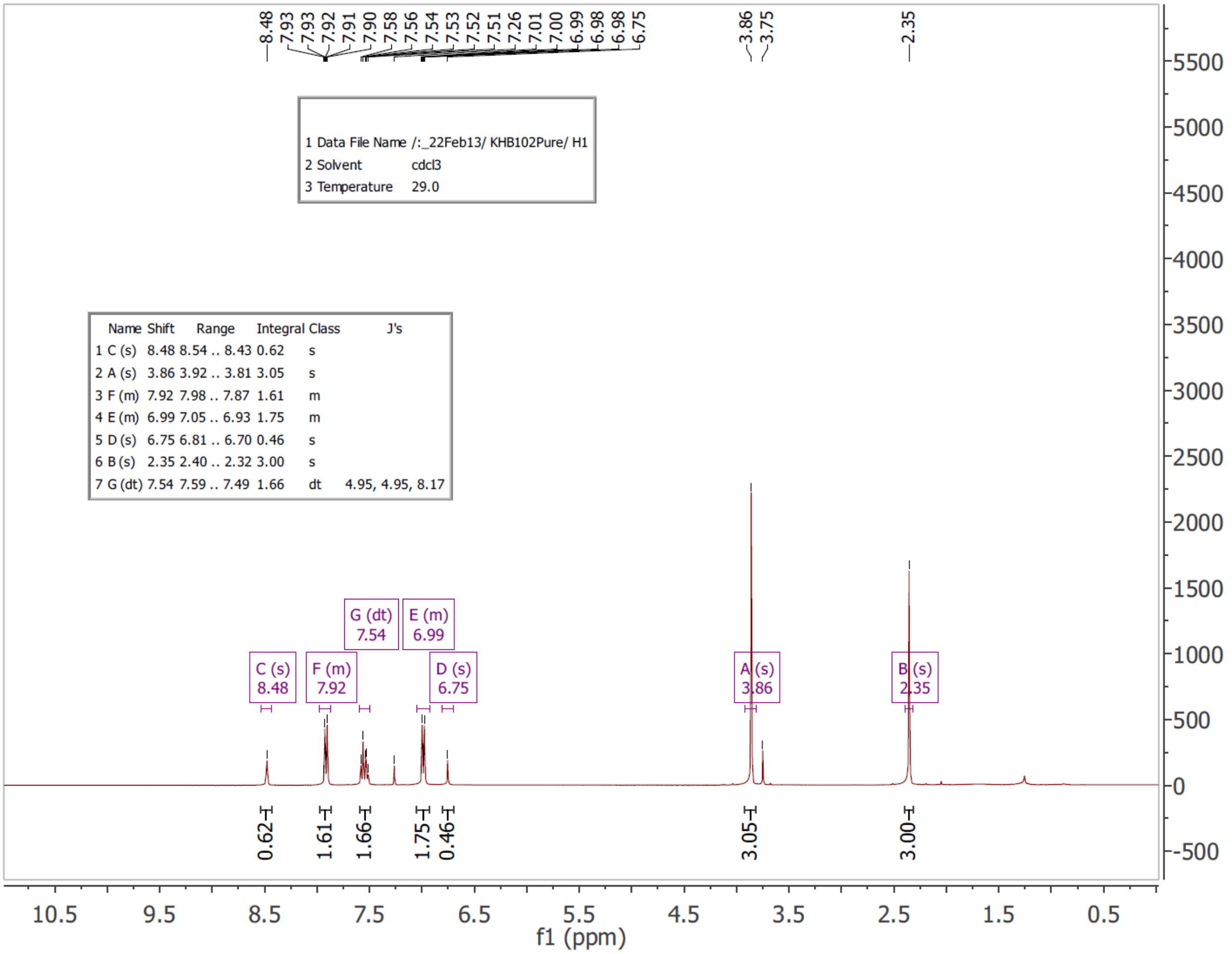

Figure **S2**. ^{1}H NMR spectrum of **2-(4'-Methoxyphenyl)-5-methylpyridine (4-MeOppy)** in $CDCl_3$

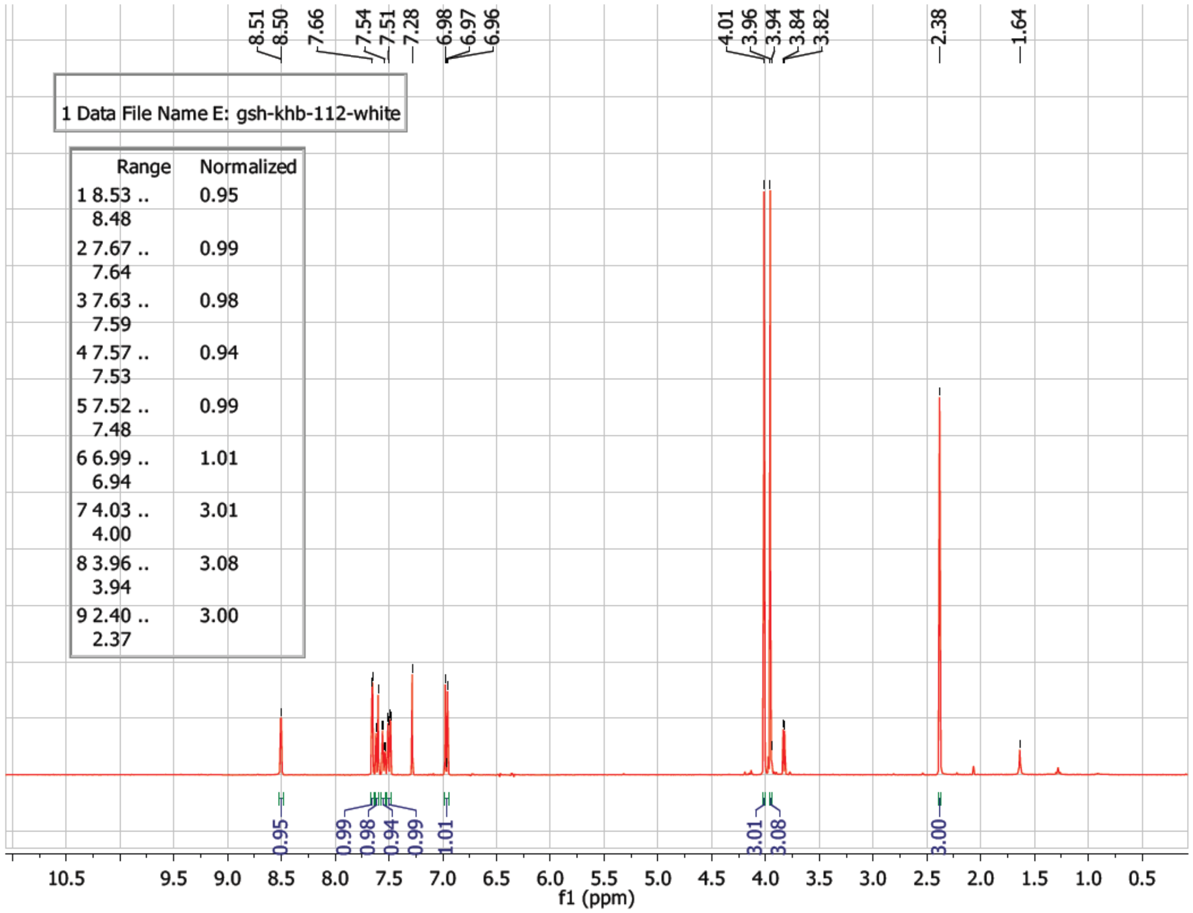

Figure **S3**. ^{1}H NMR spectrum of **2-(3',4'-Dimethoxyphenyl)-5-methylpyridine (3,4-dMeOppy)** in $CDCl_3$

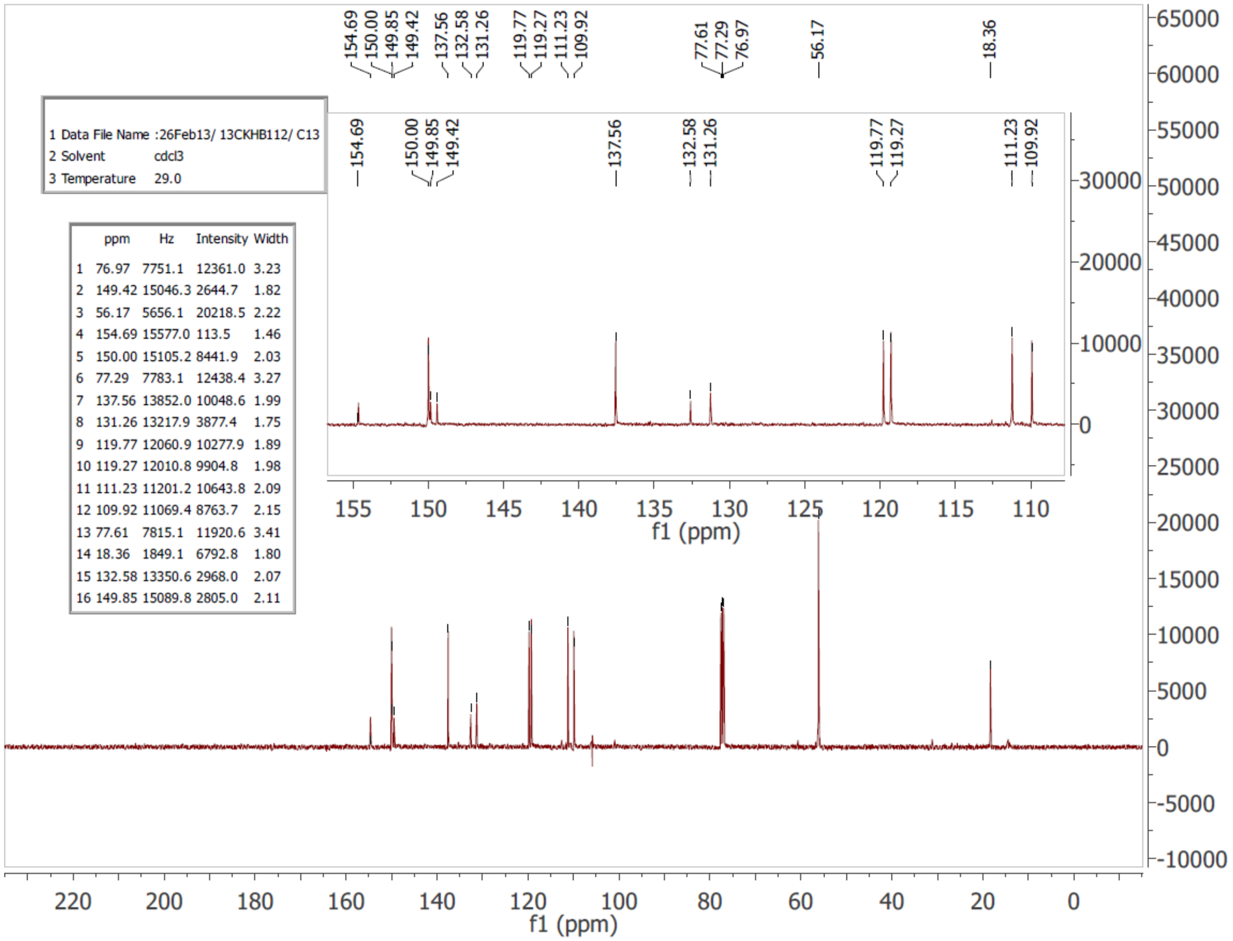

Figure **S4**. ^{13}C NMR spectrum of **2-(3',4'-Dimethoxyphenyl)-5-methylpyridine (3,4-dMeOppy)** in $CDCl_3$

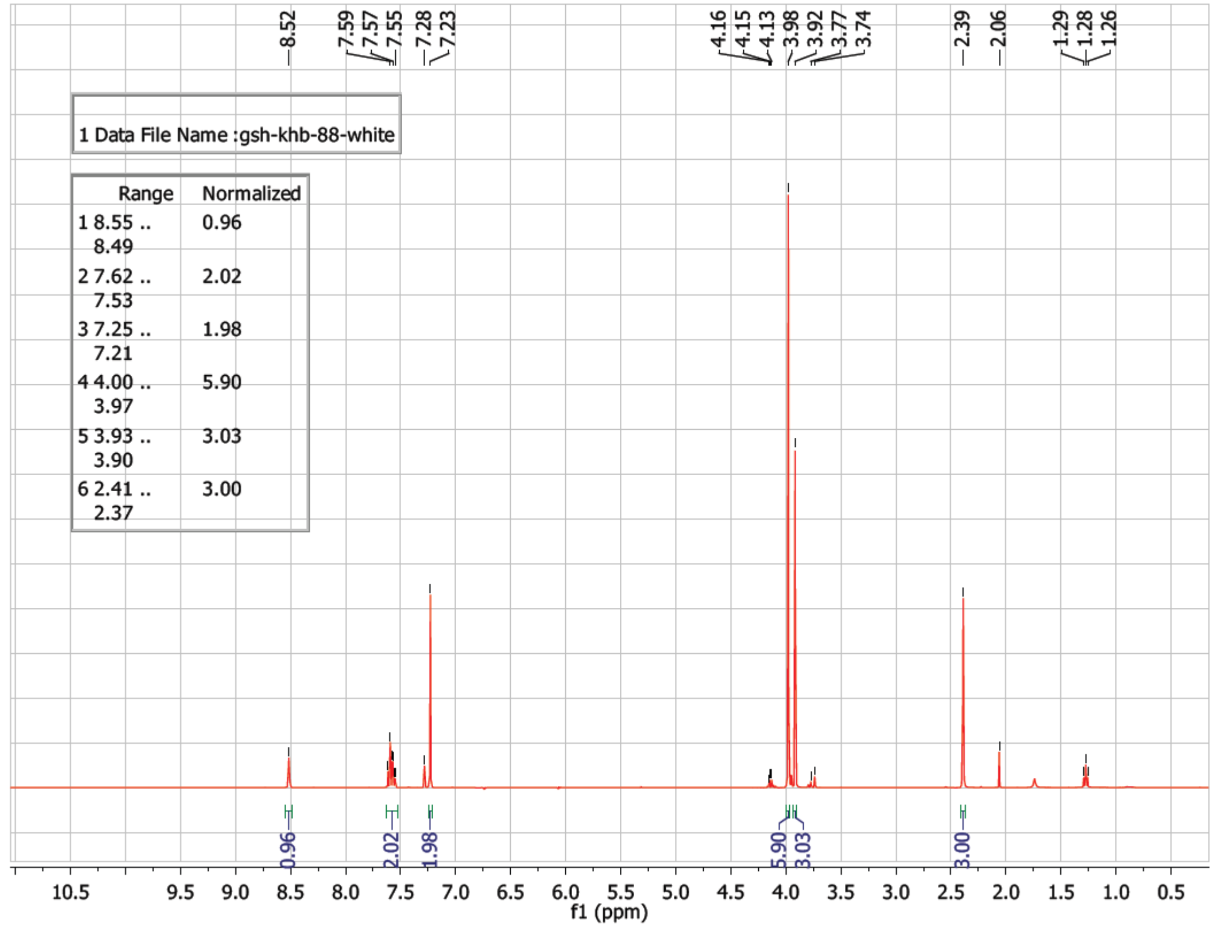

Figure **S5**. ^{1}H NMR spectrum of **2-(3',4',5'-Trimethoxyphenyl)-5-methylpyridine (3,4,5-tMeOppy)** in $CDCl_3$

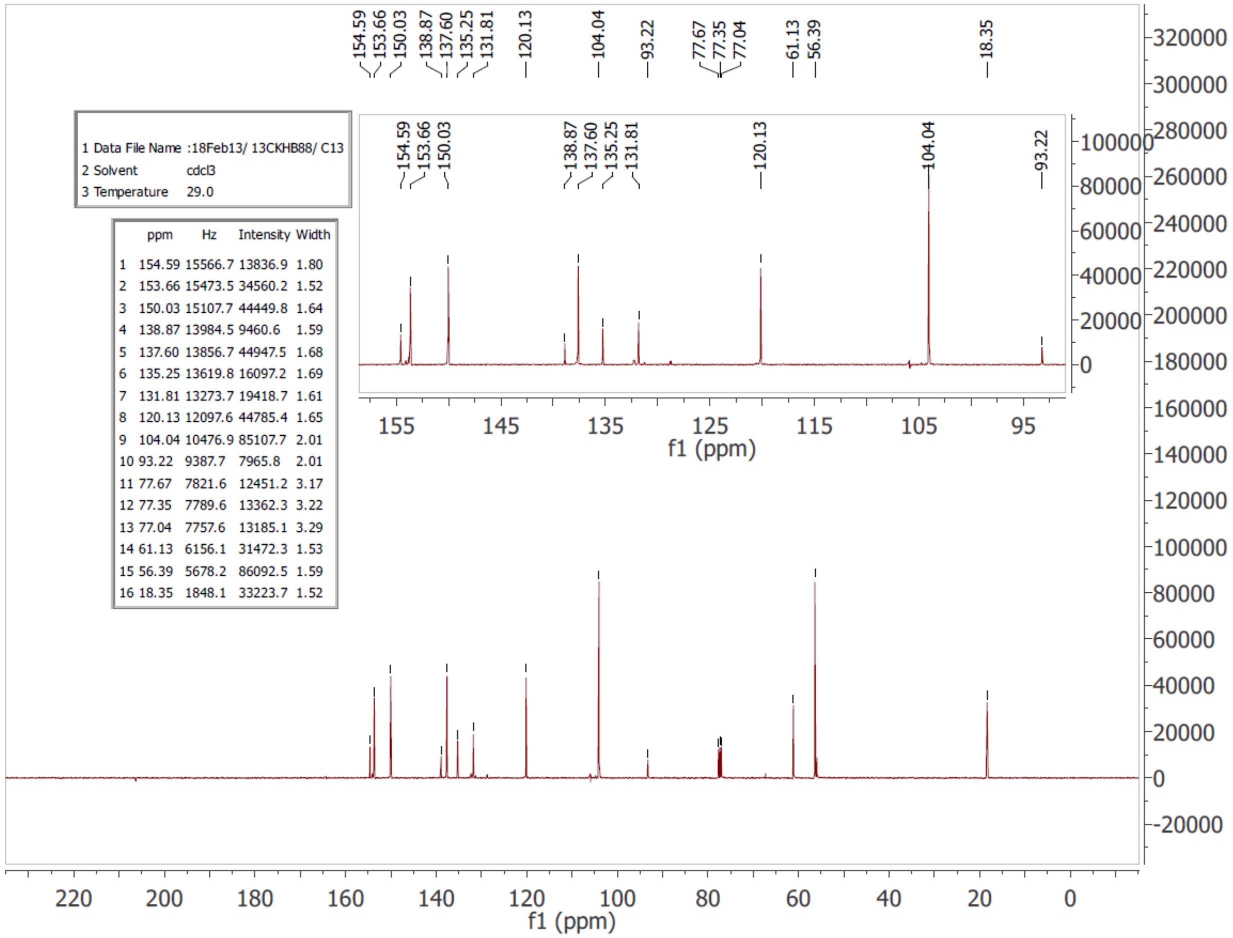

Figure **S6**. ^{13}C NMR spectrum of **2-(3’,4’,5’-Trimethoxyphenyl)-5-methylpyridine (3,4,5-tMeOppy)** in $CDCl_3$

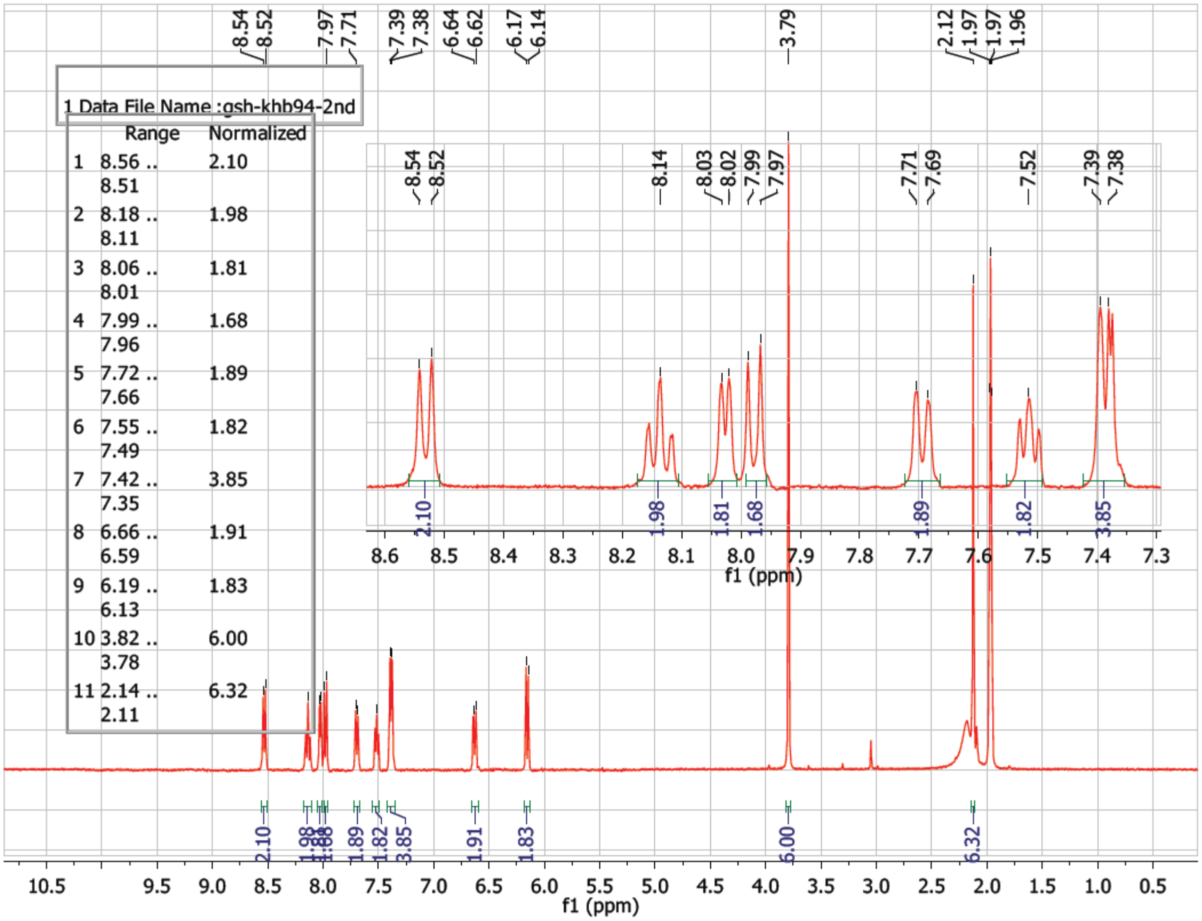

Figure **S7**. ^{1}H NMR spectrum of **[Ir(3-MeOppy)$_2$(bpy)](PF$_6$) 1a** in CD_3CN

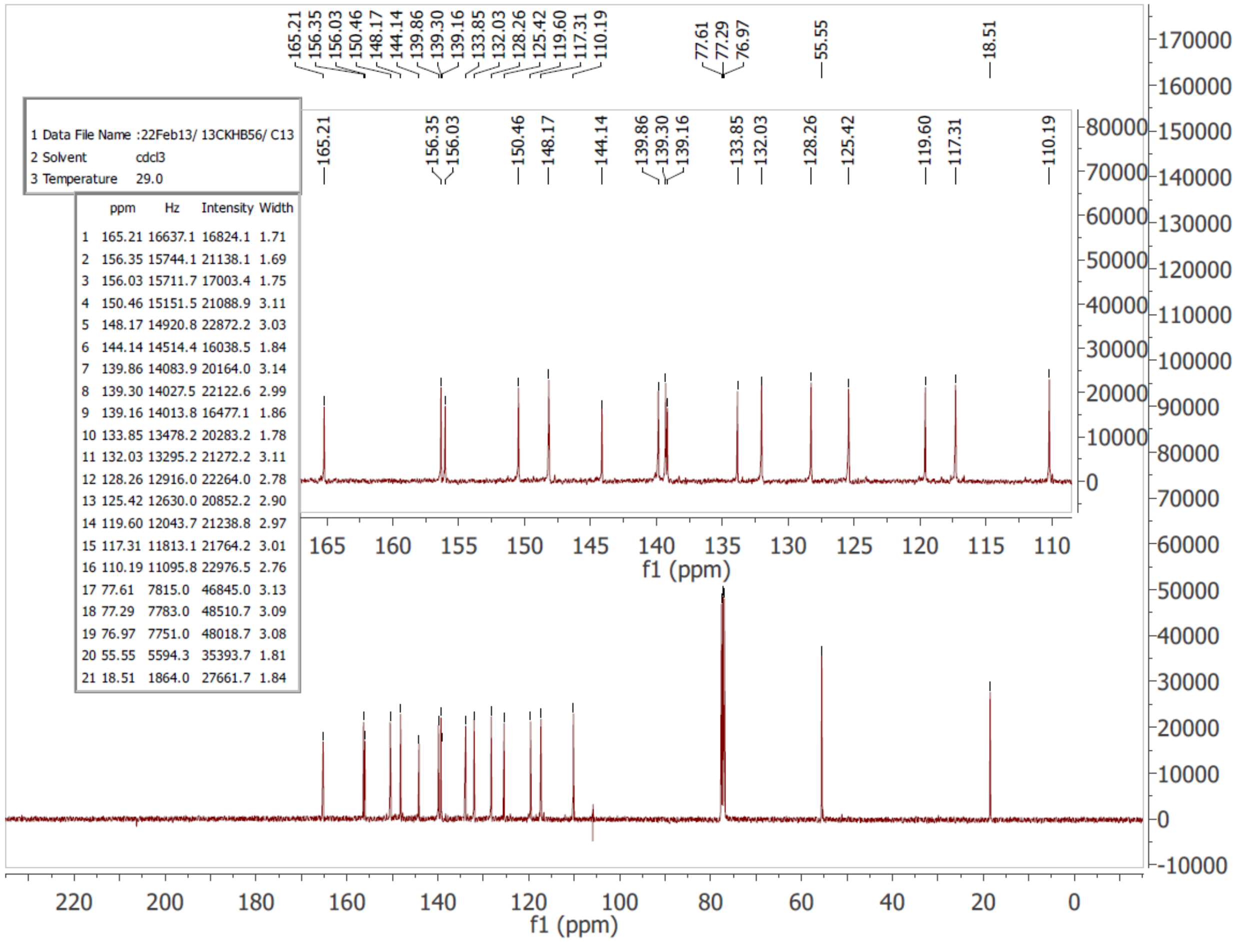

Figure **S8**. ^{13}C NMR spectrum of **[Ir(3-MeOppy)$_2$(bpy)](PF$_6$) 1a** in $CDCl_3$

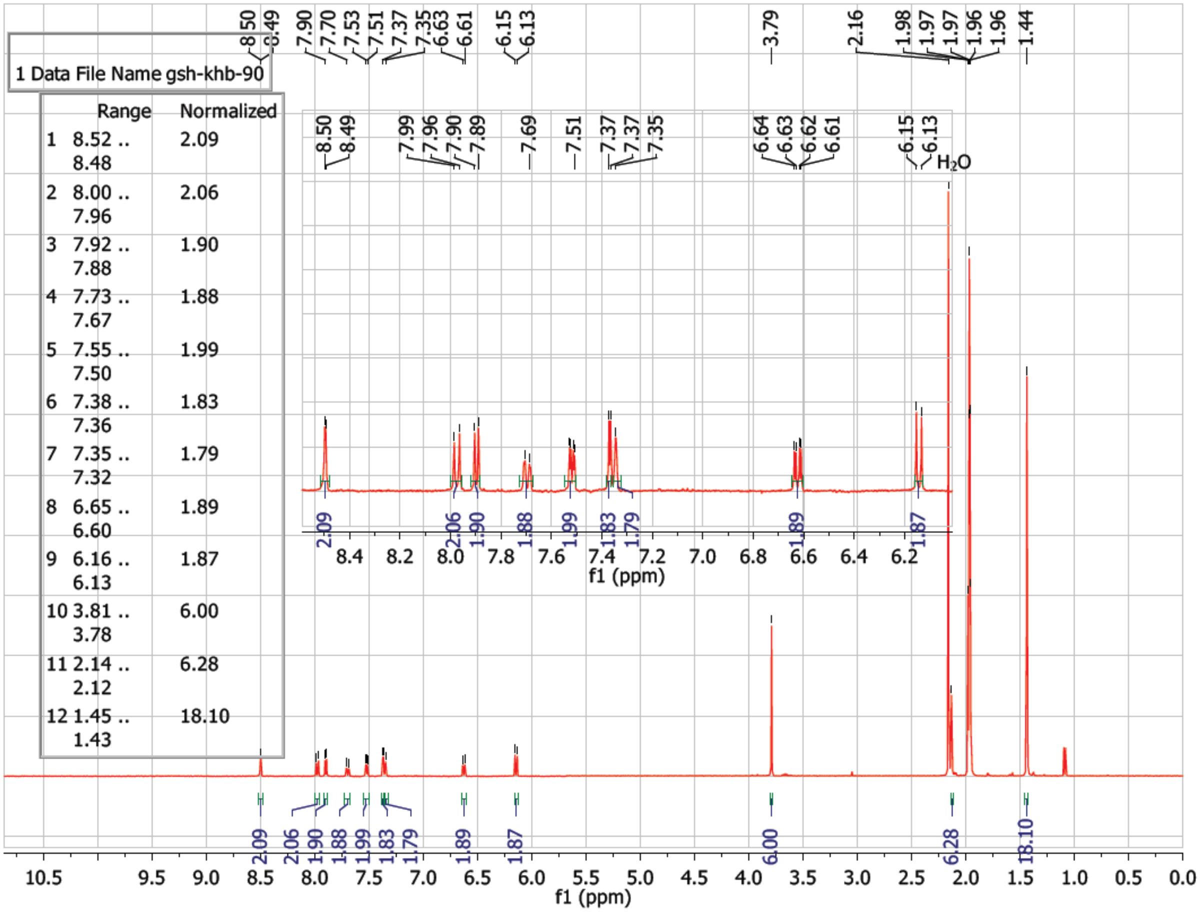

	Range	Normalized
1	8.52 .. 8.48	2.09
2	8.00 .. 7.96	2.06
3	7.92 .. 7.88	1.90
4	7.73 .. 7.67	1.88
5	7.55 .. 7.50	1.99
6	7.38 .. 7.36	1.83
7	7.35 .. 7.32	1.79
8	6.65 .. 6.60	1.89
9	6.16 .. 6.13	1.87
10	3.81 .. 3.78	6.00
11	2.14 .. 2.12	6.28
12	1.45 .. 1.43	18.10

Figure **S9**. ^{1}H NMR spectrum of **[Ir(3-MeO-ppy)$_2$(d*t*bubpy)](PF$_6$) 1b** in CD_3CN

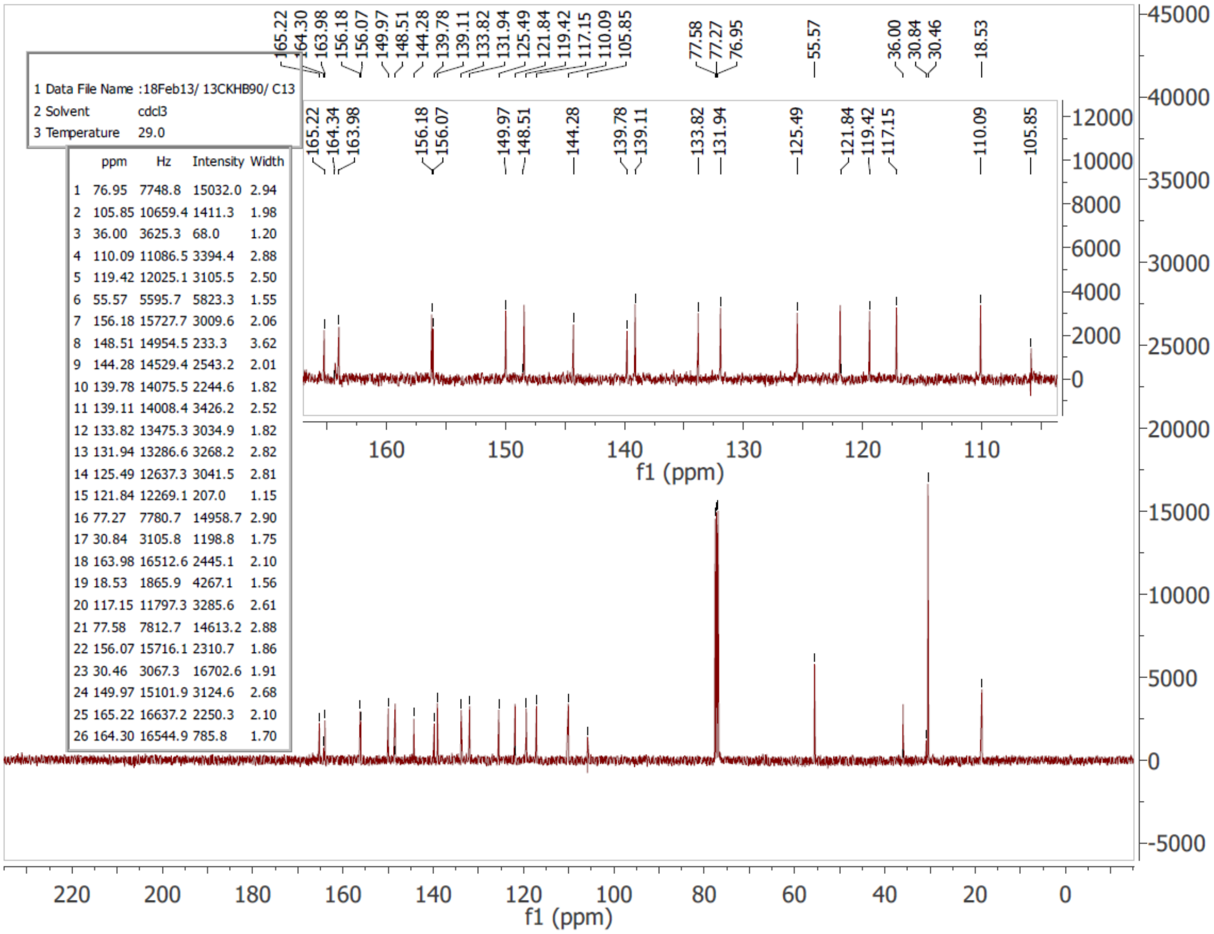

Figure **S10**. ^{13}C NMR spectrum of **[Ir(3-MeO-ppy)$_2$(d*t*bubpy)](PF$_6$) 1b** in $CDCl_3$

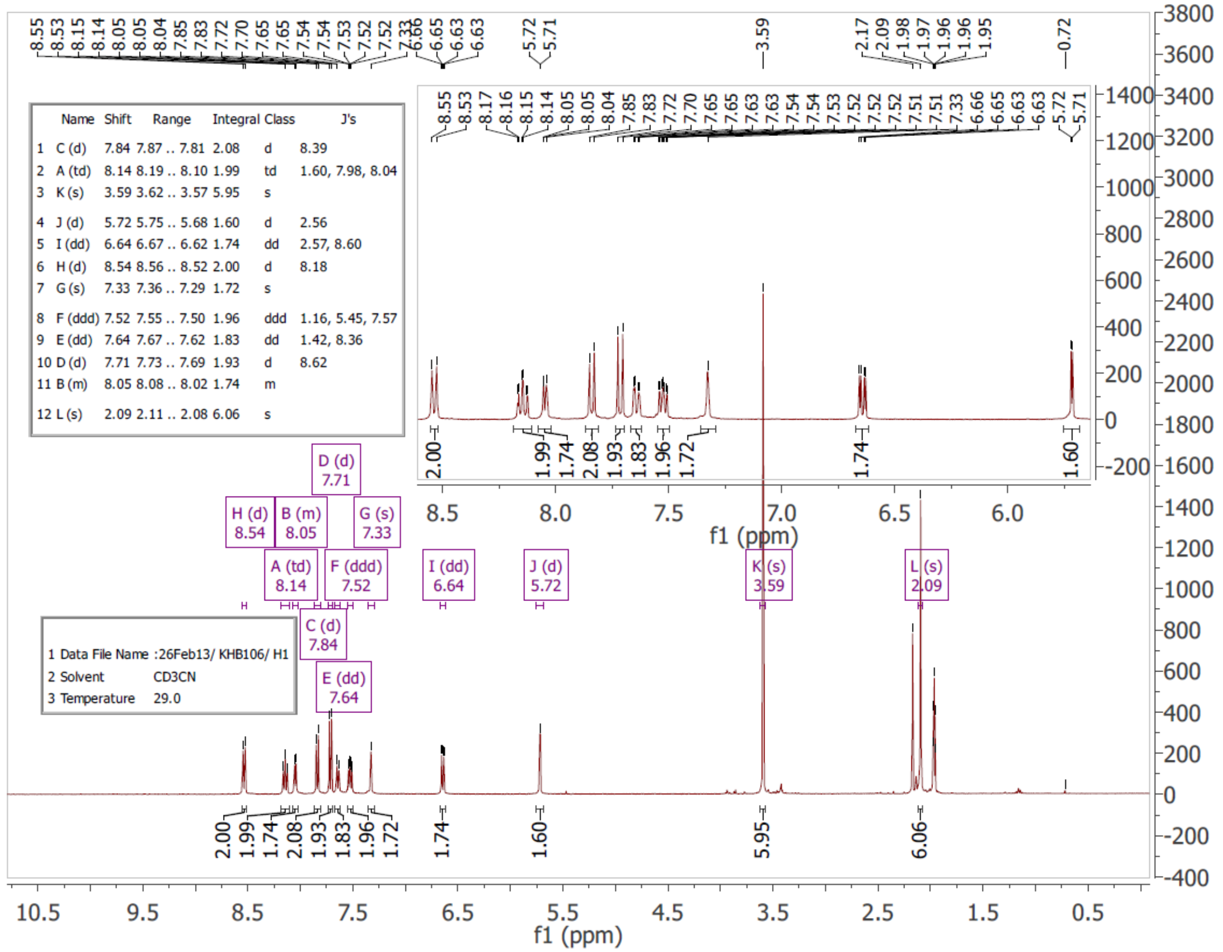

Figure **S11**. ^{1}H NMR spectrum of **[Ir(4-MeOppy)$_2$(bpy)](PF$_6$) 2a** in CD_3CN

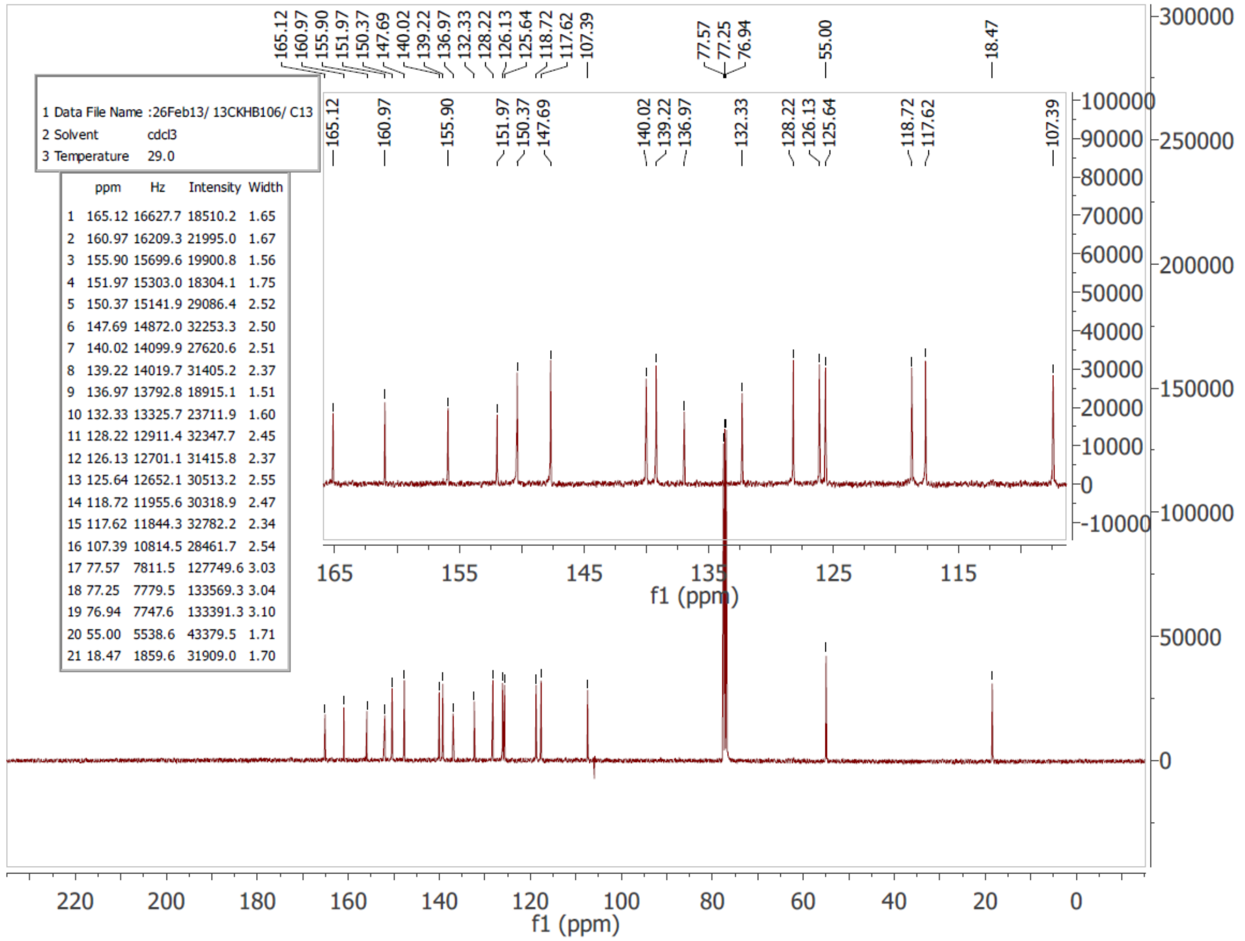

Figure **S12**. ^{13}C NMR spectrum of **[Ir(4-MeOppy)$_2$(bpy)](PF$_6$) 2a** in $CDCl_3$

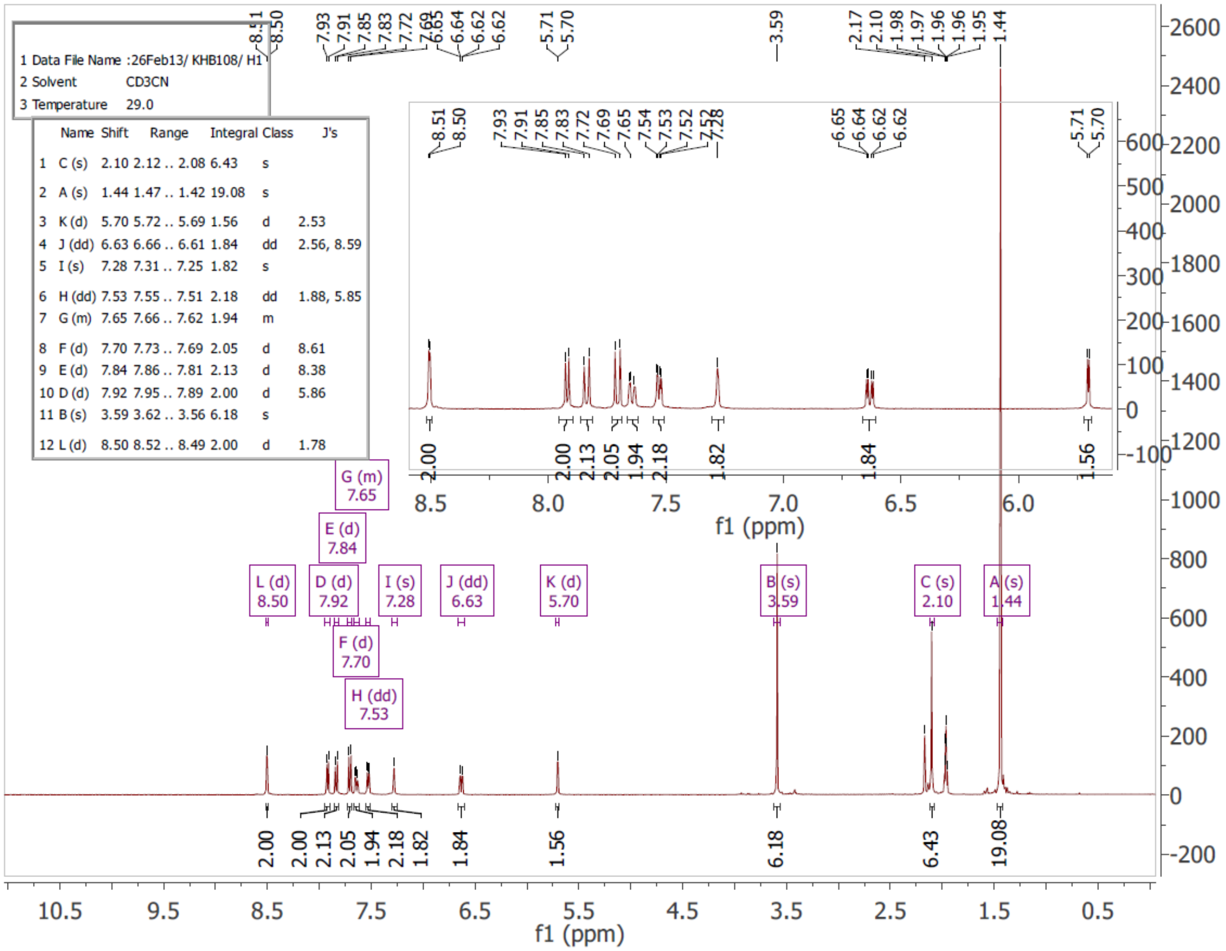

Figure **S13**. ^{1}H NMR spectrum of **[Ir(4-MeOppy)$_2$(d*t*bubpy)](PF$_6$) 2b** in CD_3CN

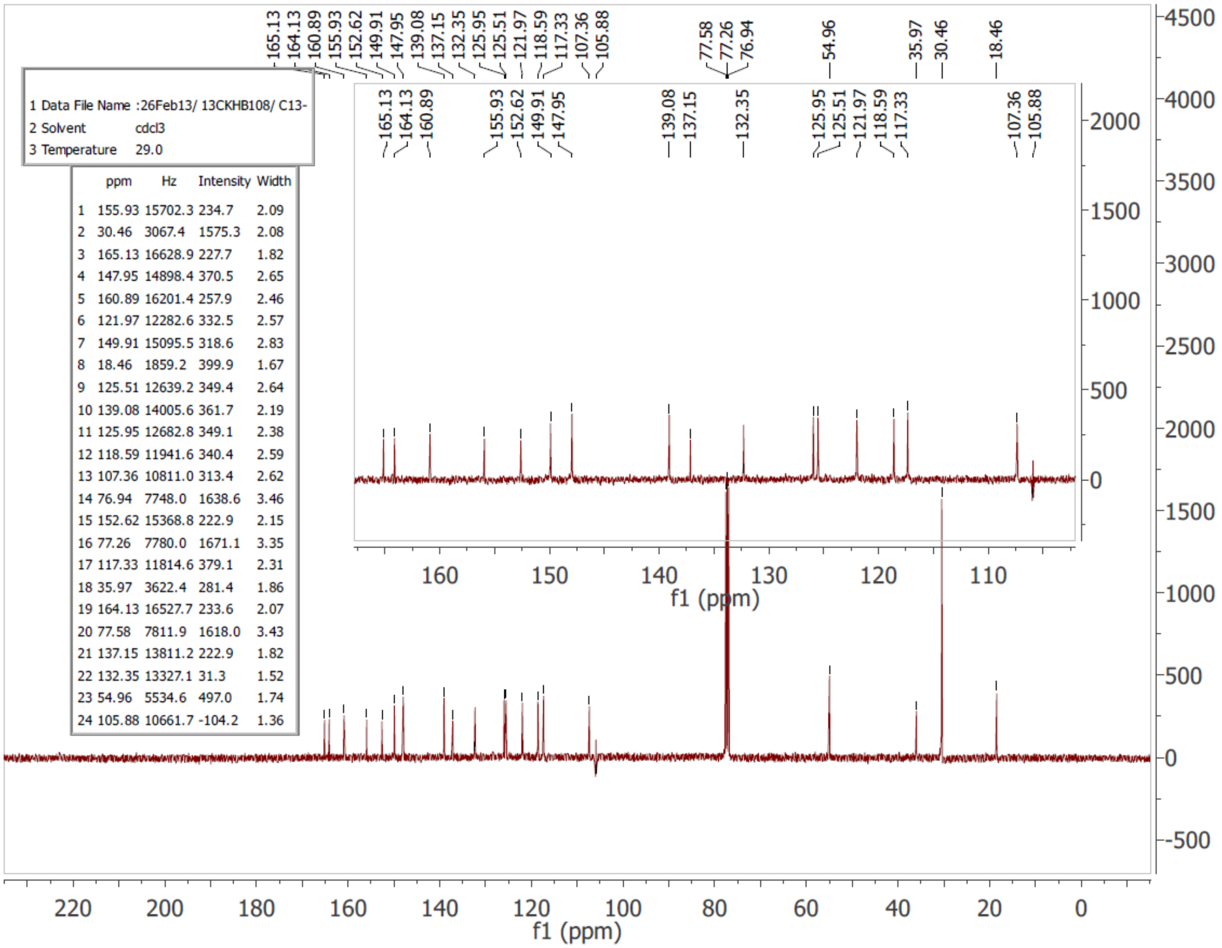

Figure **S14**. ^{13}C NMR spectrum of **[Ir(4-MeOppy)$_2$(d*t*bubpy)](PF$_6$) 2b** in $CDCl_3$

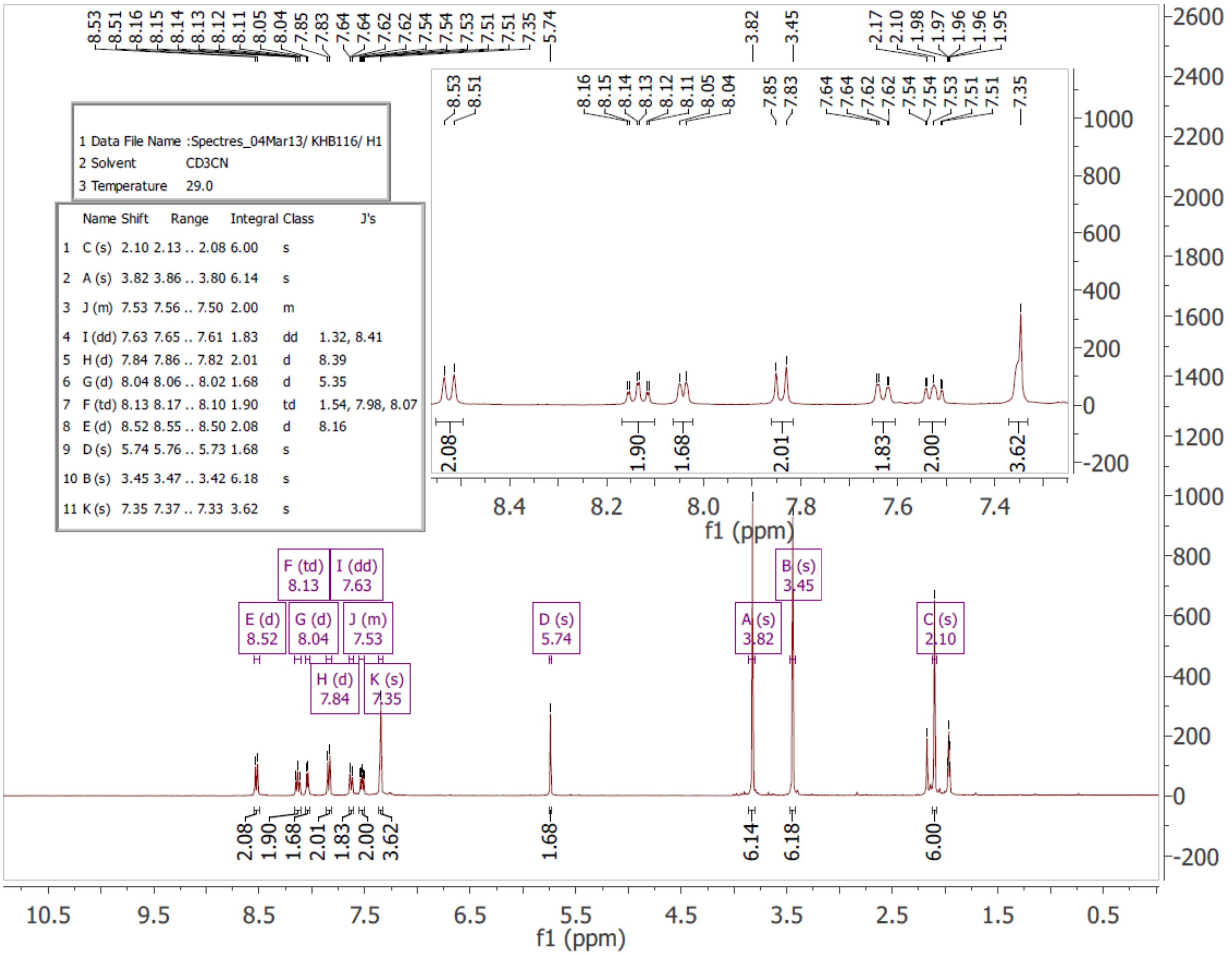

Figure **S15**. ^{1}H NMR spectrum of **[Ir(3,4-dMeOppy)$_2$(bpy)](PF$_6$) 3a** in CD_3CN

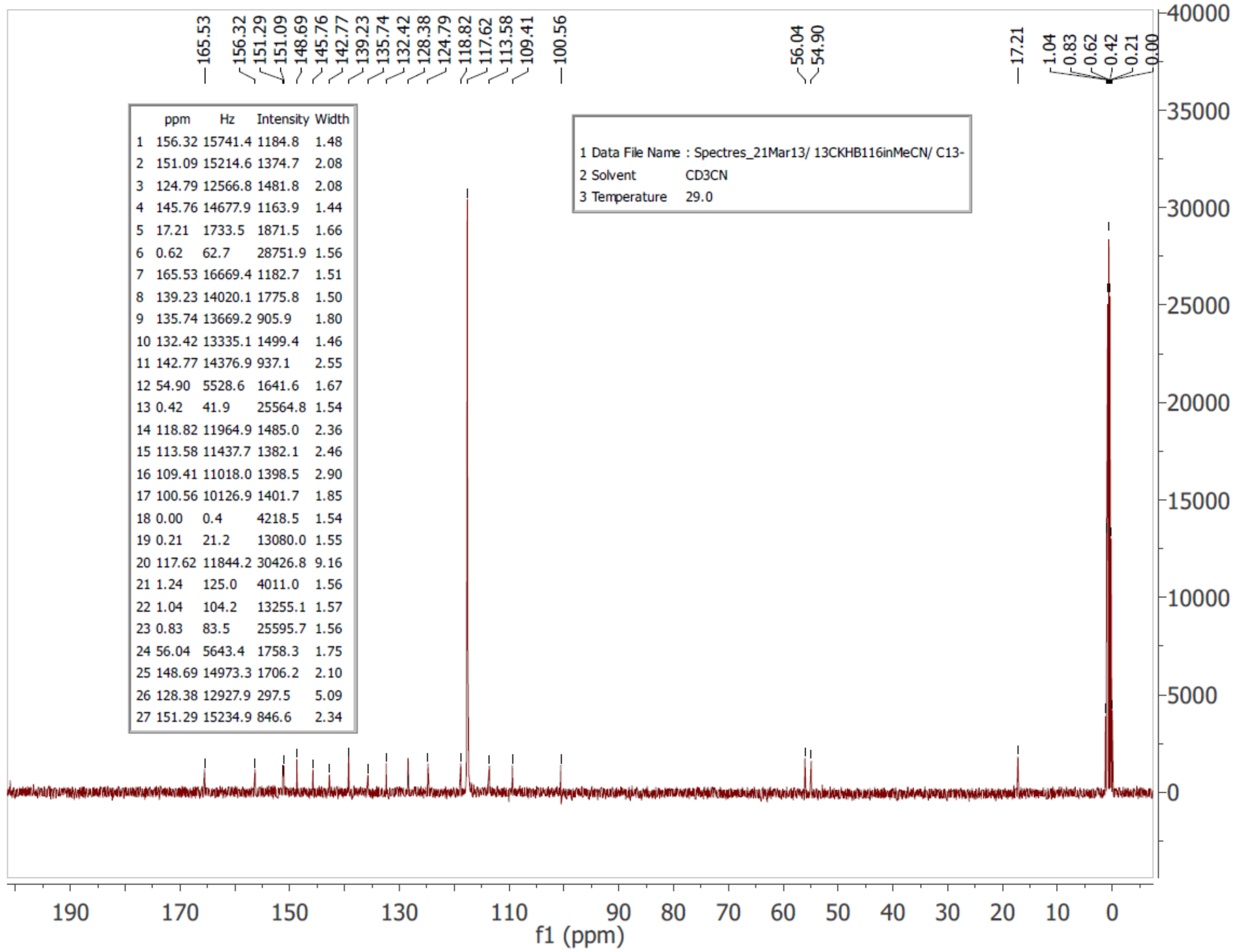

Figure **S16**. ^{13}C NMR spectrum of **[Ir(3,4-dMeOppy)$_2$(bpy)](PF$_6$) 3a** in CD$_3$CN

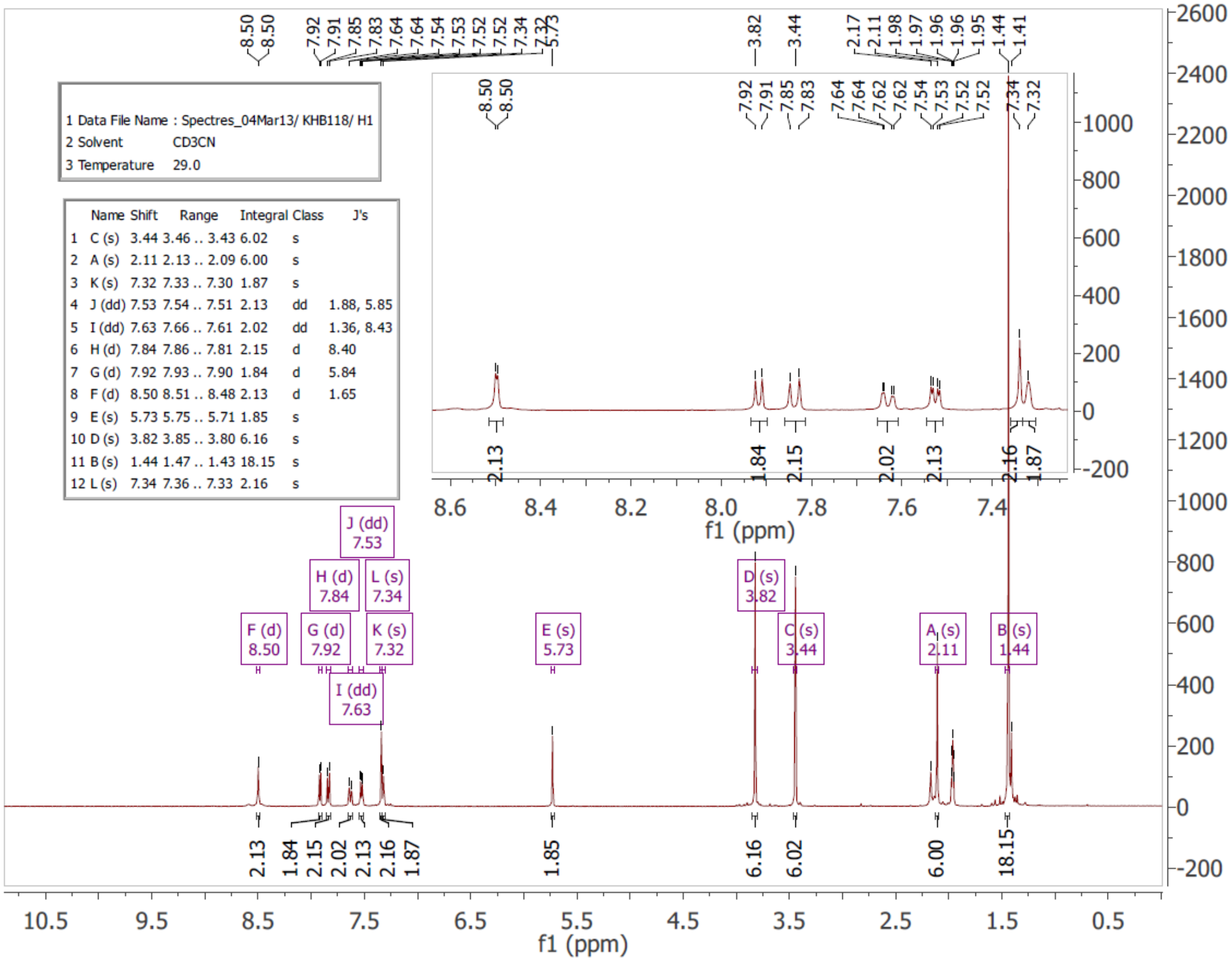

Figure **S17**. ^{1}H NMR spectrum of **[Ir(3,4-dMeOppy)$_2$(d*t*bubpy)](PF$_6$) 3b** in CD_3CN

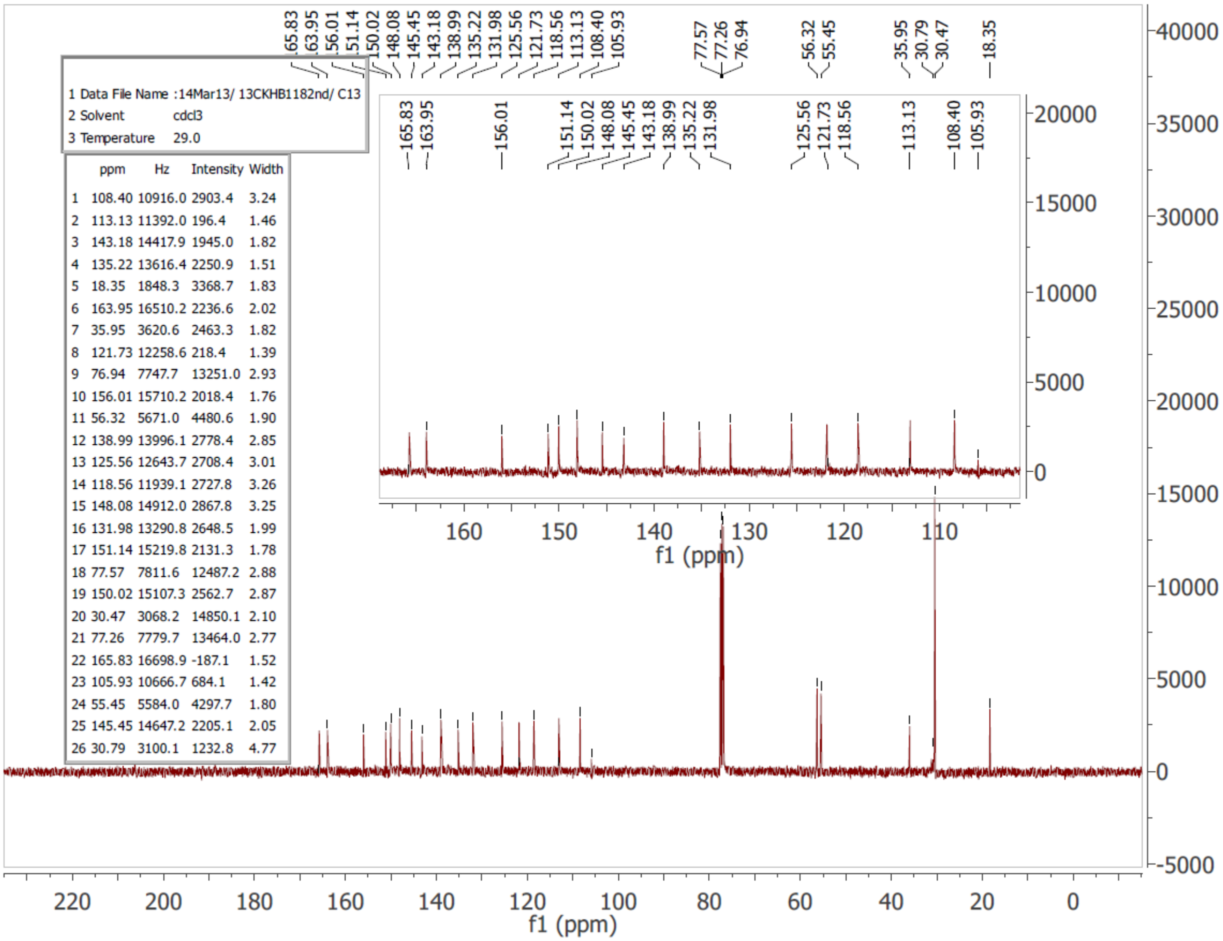

Figure **S18**. ^{13}C NMR spectrum of **[Ir(3,4-dMeOppy)$_2$(d*t*bubpy)](PF$_6$) 3b** in $CDCl_3$

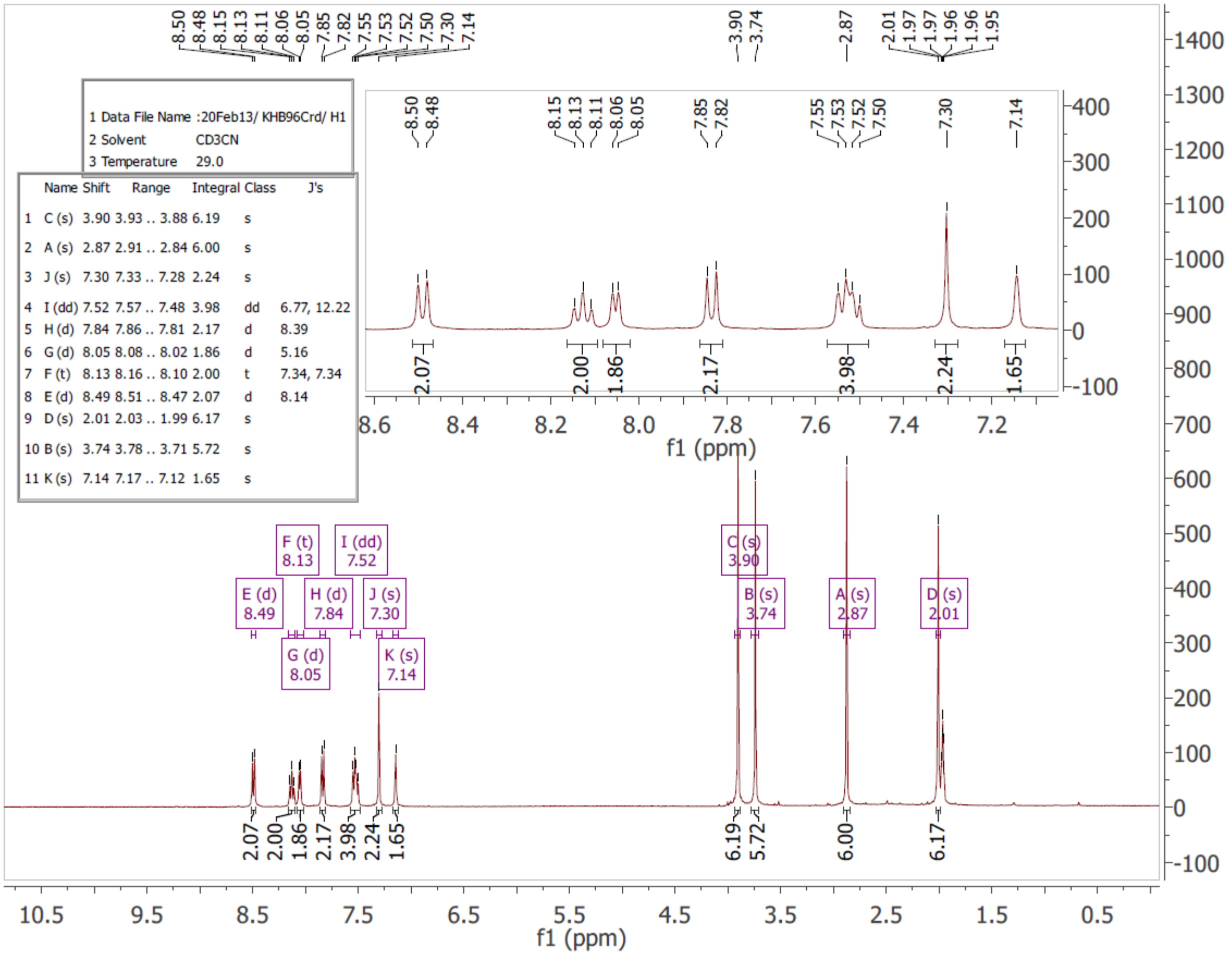

Figure **S19**. ^{1}H NMR spectrum of **[Ir(3,4,5-tMeOppy)$_2$(bpy)](PF$_6$) 4a** in CD_3CN

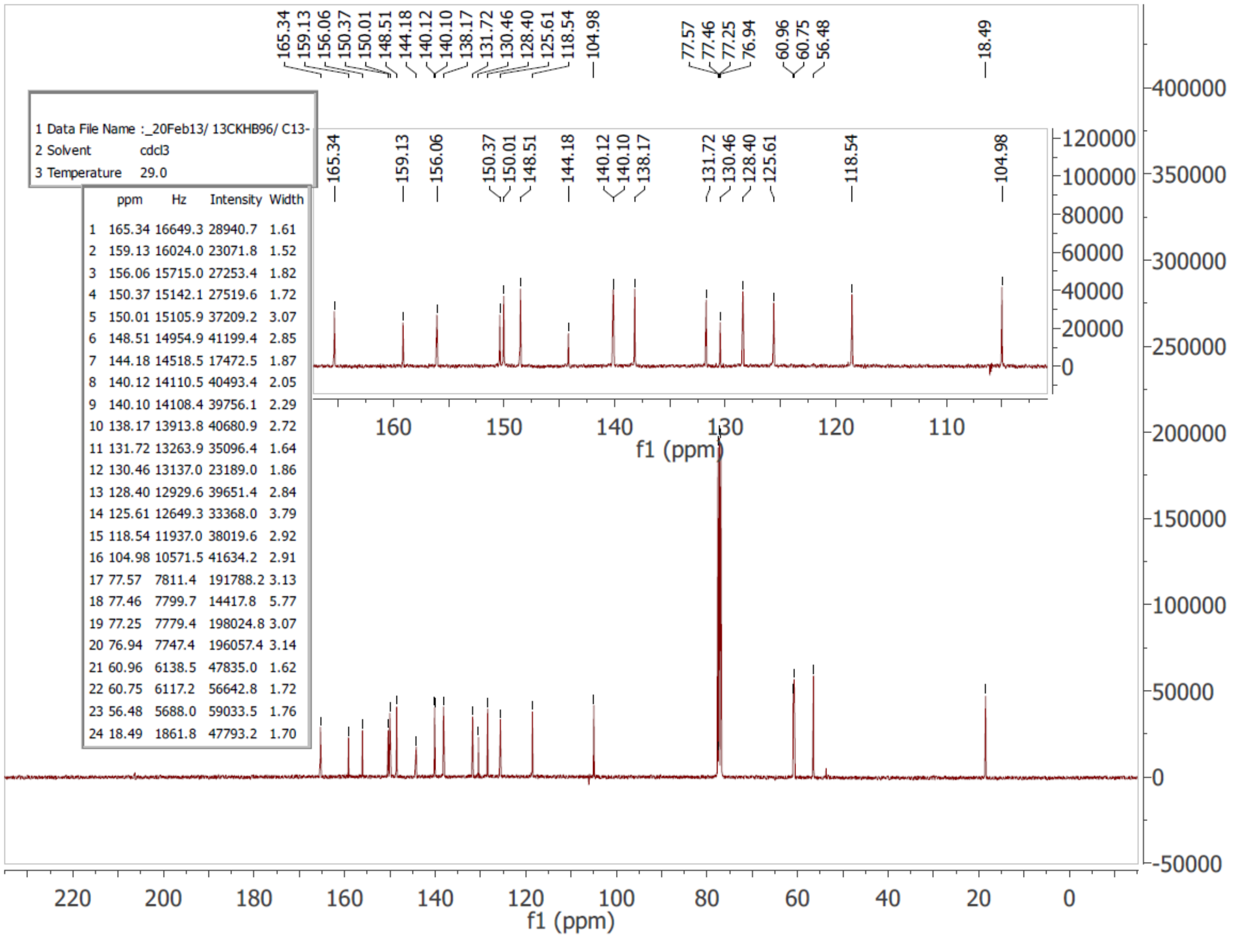

	ppm	Hz	Intensity	Width
1	165.34	16649.3	28940.7	1.61
2	159.13	16024.0	23071.8	1.52
3	156.06	15715.0	27253.4	1.82
4	150.37	15142.1	27519.6	1.72
5	150.01	15105.9	37209.2	3.07
6	148.51	14954.9	41199.4	2.85
7	144.18	14518.5	17472.5	1.87
8	140.12	14110.5	40493.4	2.05
9	140.10	14108.4	39756.1	2.29
10	138.17	13913.8	40680.9	2.72
11	131.72	13263.9	35096.4	1.64
12	130.46	13137.0	23189.0	1.86
13	128.40	12929.6	39651.4	2.84
14	125.61	12649.3	33368.0	3.79
15	118.54	11937.0	38019.6	2.92
16	104.98	10571.5	41634.2	2.91
17	77.57	7811.4	191788.2	3.13
18	77.46	7799.7	14417.8	5.77
19	77.25	7779.4	198024.8	3.07
20	76.94	7747.4	196057.4	3.14
21	60.96	6138.5	47835.0	1.62
22	60.75	6117.2	56642.8	1.72
23	56.48	5688.0	59033.5	1.76
24	18.49	1861.8	47793.2	1.70

Figure **S20**. ^{13}C NMR spectrum of **[Ir(3,4,5-tMeOppy)$_2$(bpy)](PF$_6$) 4a** in $CDCl_3$

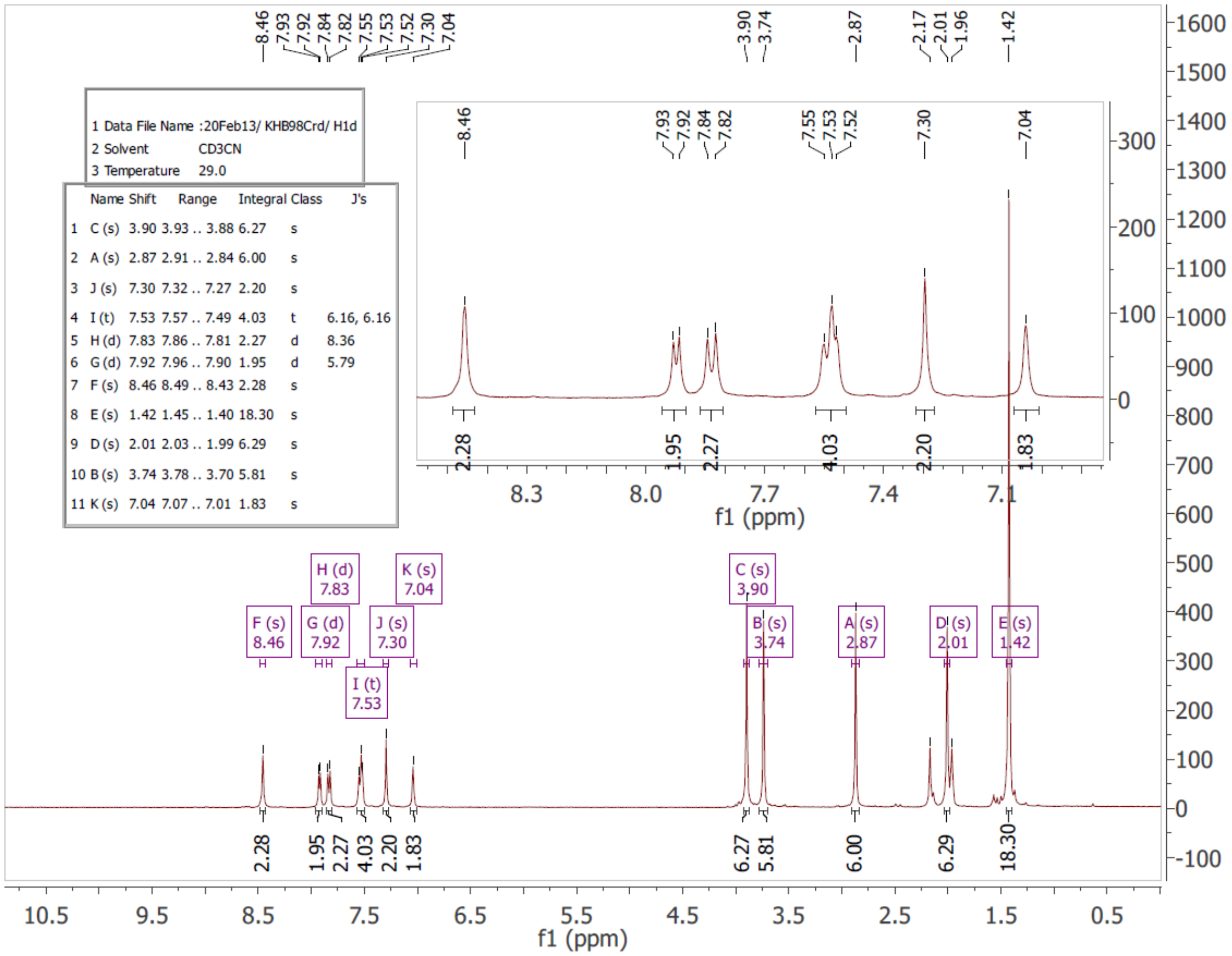

Figure **S21**. ^{1}H NMR spectrum of **[Ir(3,4,5-tMeOppy)$_2$(d*t*bubpy)](PF$_6$) 4b** in CD_3CN

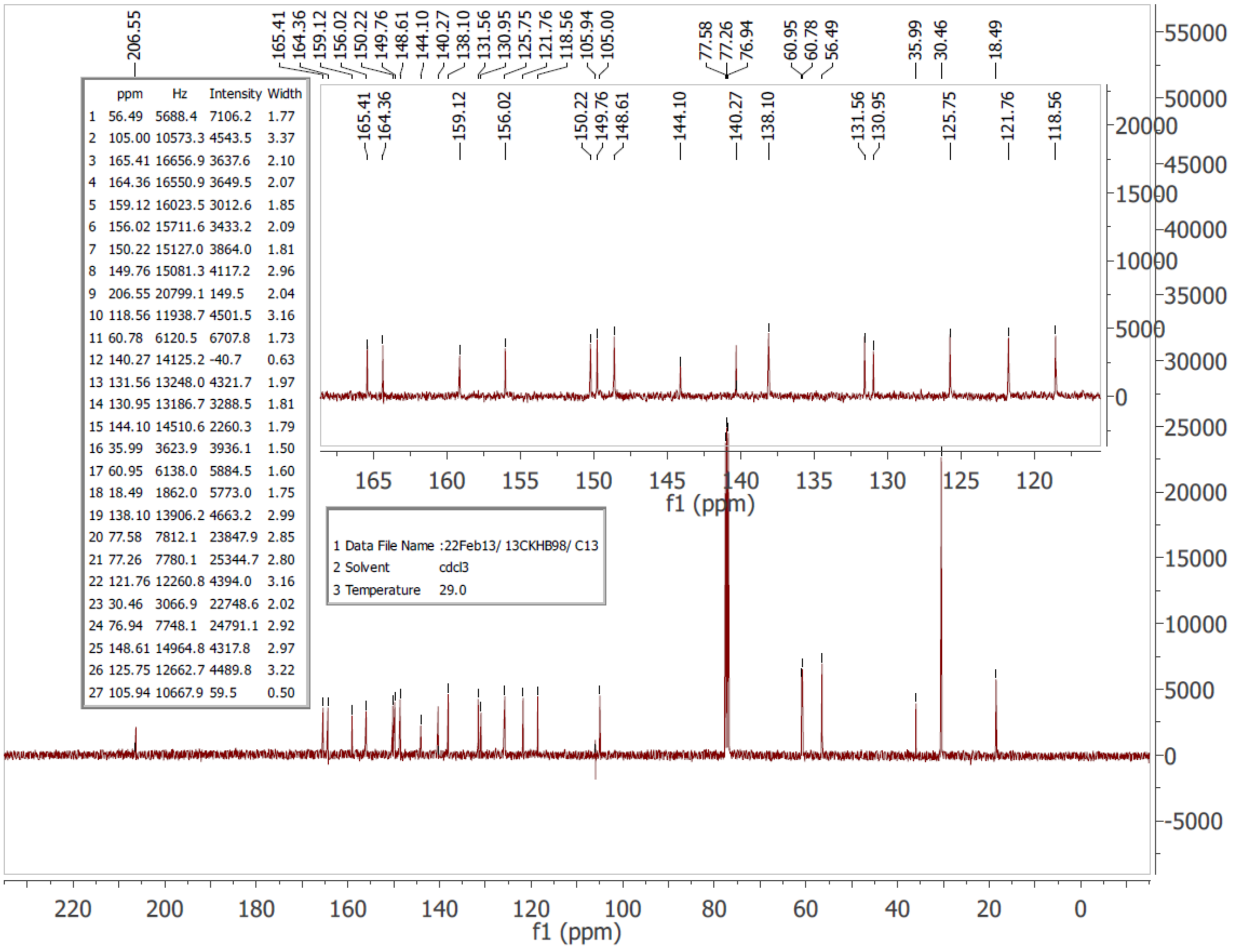

Figure **S22**. ^{13}C NMR spectrum of **[Ir(3,4,5-tMeOppy)$_2$(d*t*bubpy)](PF$_6$) 4b** in $CDCl_3$

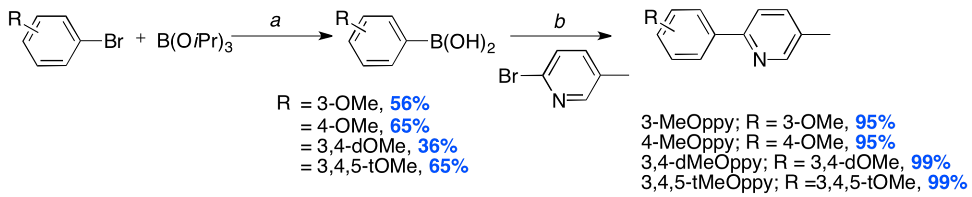

Figure **S23**. Synthesis of C^N ligands. Reagents and conditions: [a] i. toluene/THF (4:1 v/v), 1.2 equiv. *n*-BuLi, -78 °C, N_2; ii. 1 N HCl, -20 °C, RT, 0.5 h. [b] 1,4-dioxane/H_2O (4:1 v/v), 3.0 equiv. K_2CO_3, 5 mol% $Pd(PPh_3)_4$, N_2, 105 °C, 19 h.

Table **S1**. Relevant electrochemical data for complexes **1a-4b**.[a]

complex	ligands		$E_{1/2,OX}$	ΔE_p	$E_{1/2,red}$	ΔE_p	ΔE
	C^N	N^N	V	mV	V	mV	V
**1a**	3-MeOppy	bpy	0.95	76	-1.40	69	2.38
**1b**	3-MeOppy	d*t*Bubpy	0.90	75	-1.52	70	2.49
**2a**	4-MeOppy	bpy	1.15[b]	-	-1.42	70	2.61
**2b**	4-MeOppy	d*t*Bubpy	1.13[b]	-	-1.49	70	2.63
**3a**	3,4-dMeOppy	bpy	0.84	75	-1.41	71	2.27
**3b**	3,4-dMeOppy	d*t*Bubpy	0.78	69	-1.52	67	2.35
**4a**	3,4,5-tMeOppy	bpy	0.87	75	-1.40	74	2.30
**4b**	3,4,5-tMeOppy	d*t*Bubpy	0.84	76	-1.50	70	2.42

[a] CV traces recorded in MeCN solution with 0.1 M (n-$Bu_4N)PF_6$ at 298 K at 50 mVs^{-1}. Values are in V vs. SCE (Fc/Fc^+ vs. SCE = 0.38 V).[1] A non-aqueous Ag/Ag^+ electrode (silver wire in a solution of 0.1 M $AgNO_3$ in MeCN) was used as the pseudoreference electrode; a glassy-carbon electrode was used for the working electrode and a Pt electrode was used as the counter electrode. [b] Irreversible and E_{pa} reported for oxidation peak potentials.

Table **S2**. Relevant spectroscopic data for complex **1a-4b**.

Complex	λ_{abs} / nm (ε /10^4 M^{-1} cm^{-1})[a]	$E_{0,0}$ (nm)
**1a**	265 (8.37), 311 (3.14), 338 (1.61), 425 (0.70)	498
**1b**	263 (10.61), 308 (4.18), 338 (2.09), 425(0.80)	492
**2a**	255 (5.32), 277 (6.78), 309 (3.71), 390 (0.86), 450* (0.11)	464
**2b**	250 (6.62), 278 (8.88), 310 (3.75), 390 (0.82), 450* (0.09)	471
**3a**	279 (5.72), 308 (2.47), 340 (1.57), 419 (0.74)	472
**3b**	279 (6.76), 312 (2.75), 343 (1.78), 424 (0.78)	480
**4a**	244 (6.22), 275 (6.68), 310 (4.21), 330 (2.72), 385 (0.93)	460
**4b**	248 (8.81), 275 (9.45), 310 (5.89), 331 (3.76), 395 (1.14)	473

[a] Absorption spectra recorded in aerated MeCN at 298 K. Molar absorptivities (e) determined over a concentration range of 6.88 x 10^{-1} to 3.19 x 10^{2} μM wherein absorbance values obey the Beer-Lambert law. [b] $E_{0,0}$ was estimated from the onset of the absorption spectrum at approximately 10% intensity. * Shoulder.

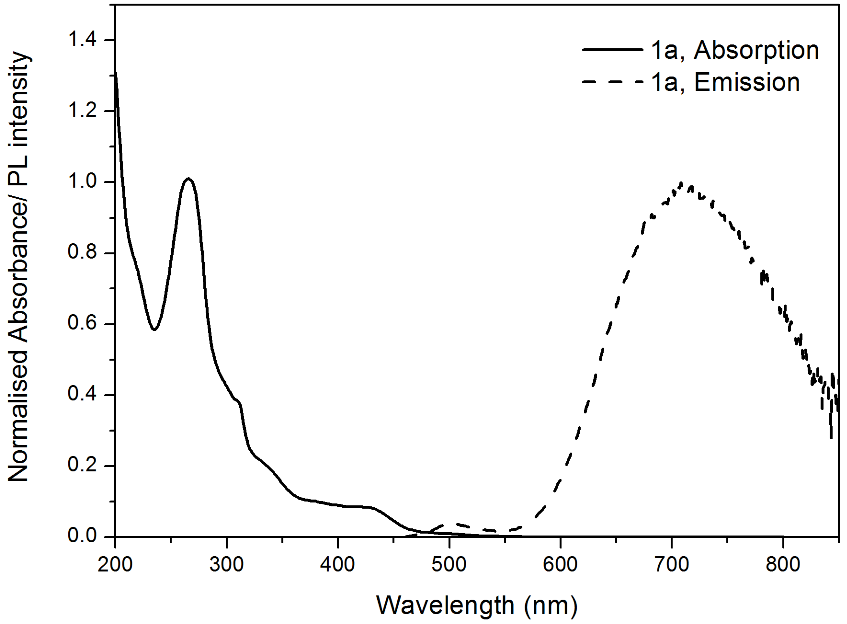

Figure **S23**. Normalized absorption and emission spectra of **[Ir(3-MeOppy)$_2$(bpy)](PF$_6$) 1a** recorded in MeCN at 298 K

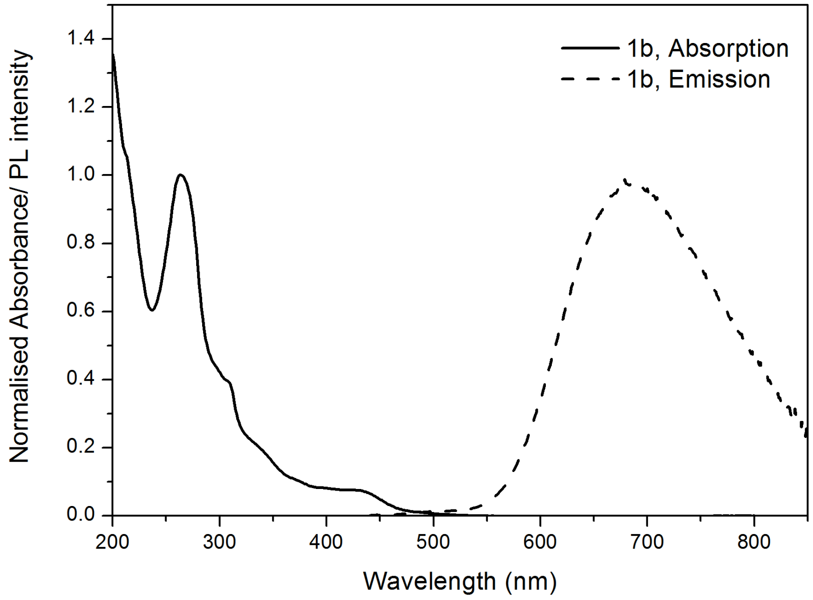

Figure **S24**. Normalized absorption and emission spectra of **[Ir(3-MeO-ppy)$_2$(d*t*bubpy)](PF$_6$) 1b** recorded in MeCN at 298 K

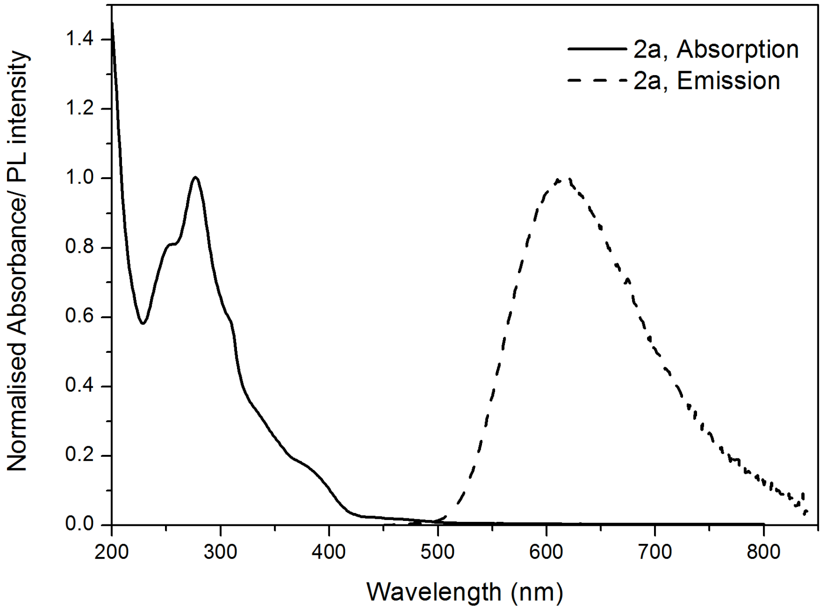

Figure **S25**. Normalized absorption and emission spectra of **[Ir(4-MeOppy)$_2$(bpy)](PF$_6$) 2a** recorded in MeCN at 298 K

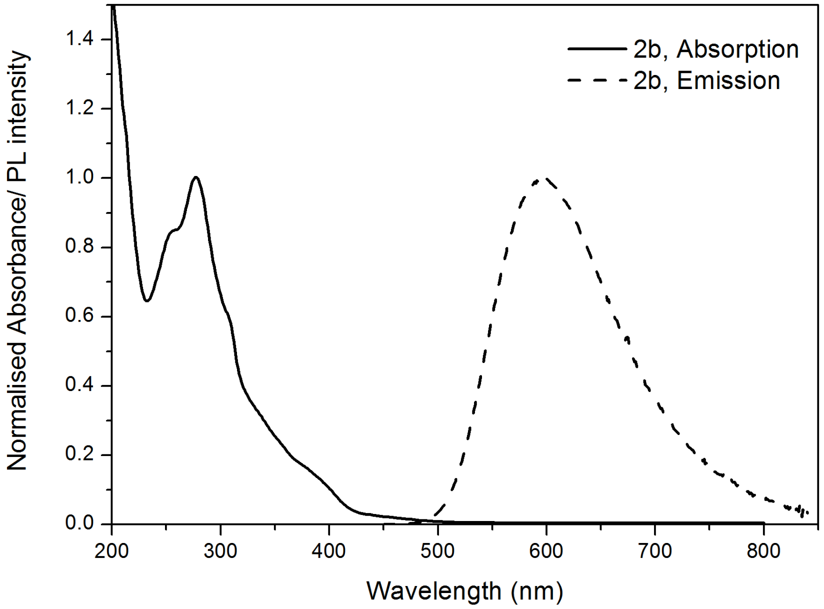

Figure **S26**. Normalized absorption and emission spectra of **[Ir(4-MeOppy)$_2$(d*t*bubpy)](PF$_6$) 2b** recorded in MeCN at 298

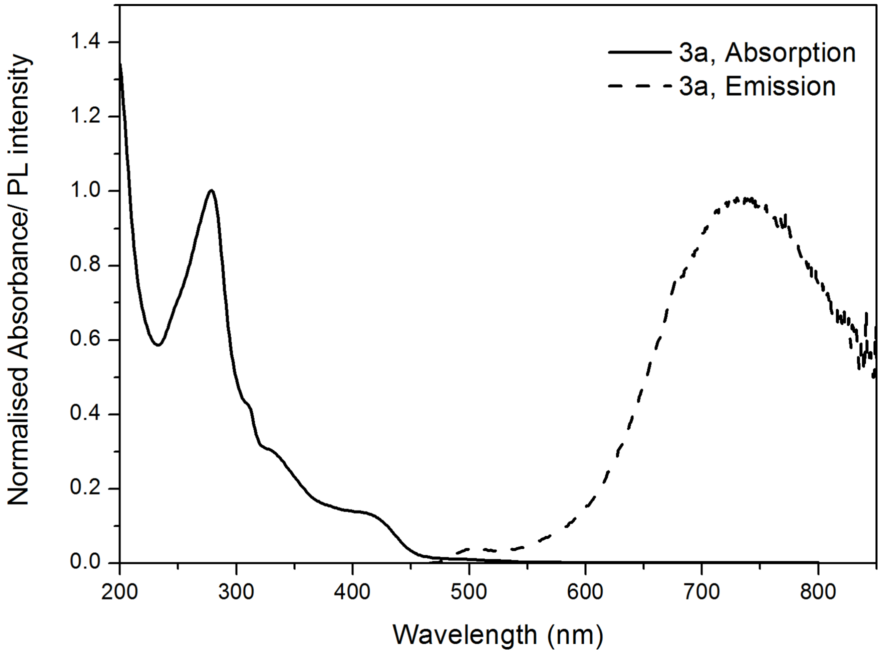

Figure **S27**. Normalized absorption and emission spectra of **[Ir(3,4-dMeOppy)$_2$(bpy)](PF$_6$) 3a** recorded in MeCN at 298 K

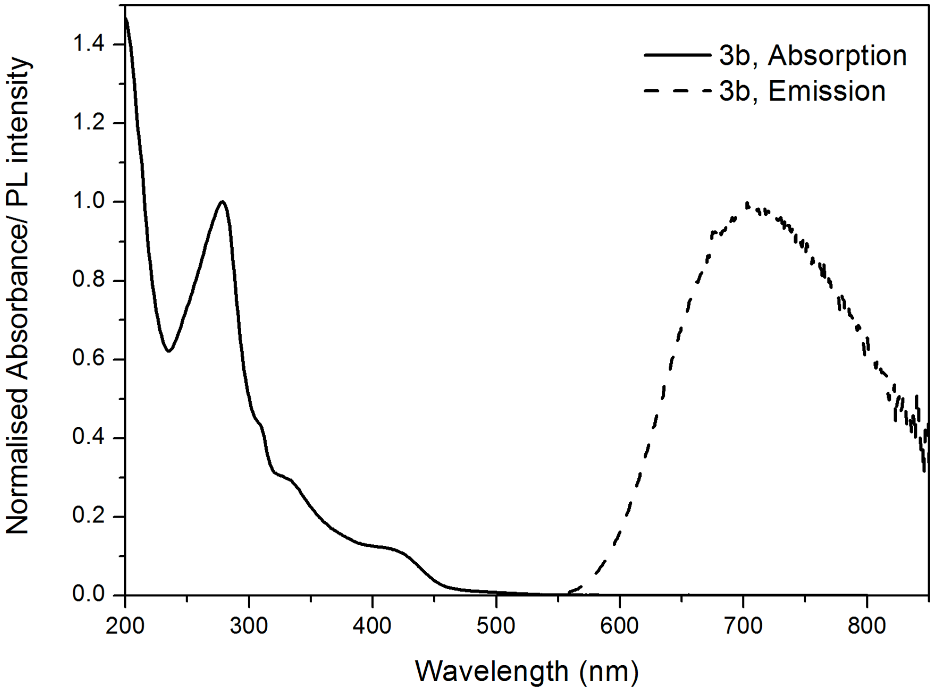

Figure **S28**. Normalized absorption and emission spectra **[Ir(3,4-dMeOppy)$_2$(d*t*bubpy)](PF$_6$) 3b** recorded in MeCN at 298 K

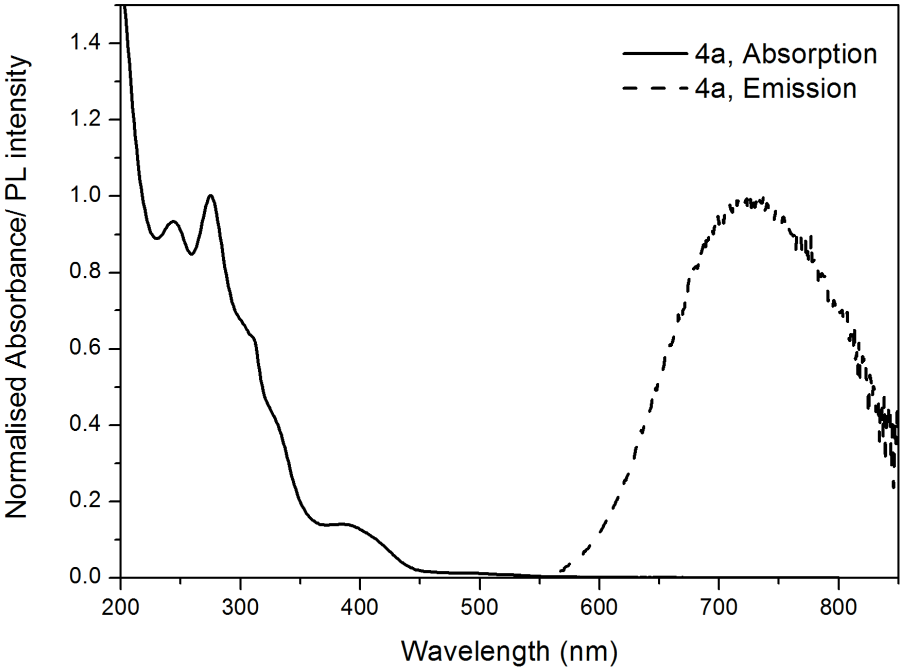

Figure **S29**. Normalized absorption and emission spectra of **[Ir(3,4,5-tMeOppy)$_2$(bpy)](PF$_6$) 4a** recorded in MeCN at 298 K

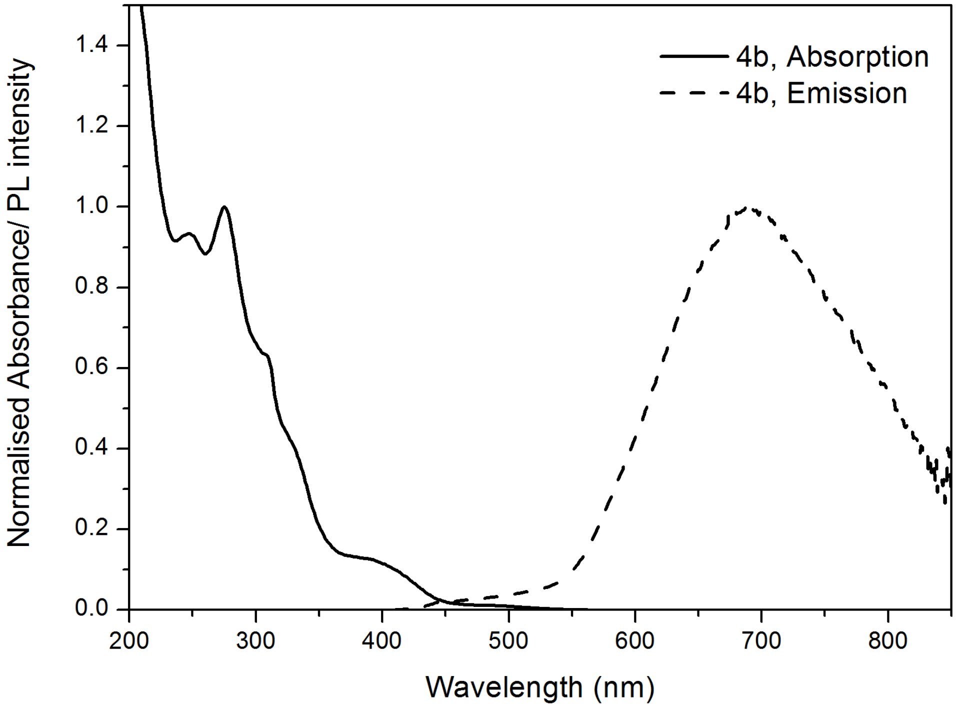

Figure **S30**. Normalized absorption and emission spectra of **[Ir(3,4,5-tMeOppy)$_2$(d*t*bubpy)](PF$_6$) 4b** recorded in MECNat 298 K

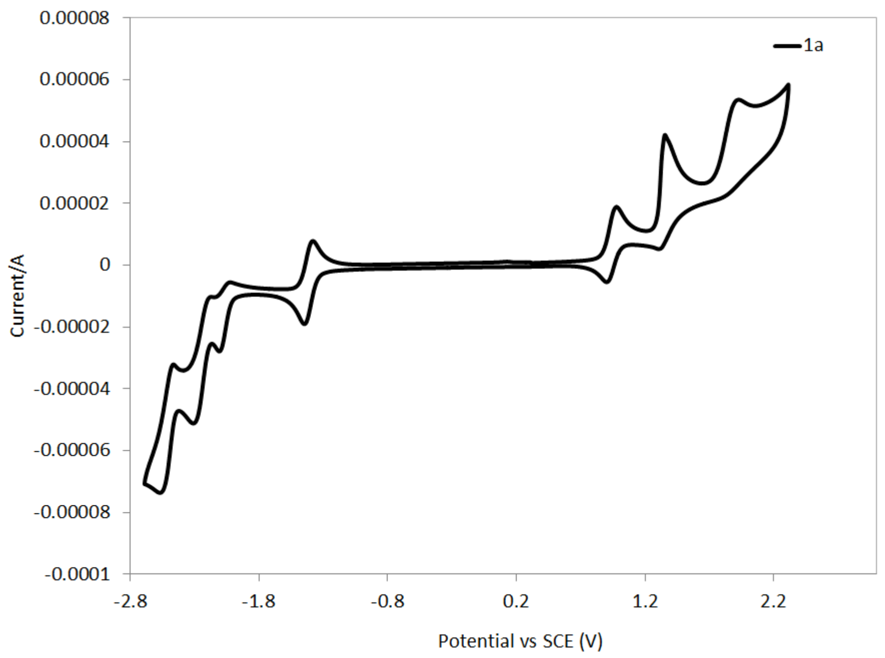

Figure **S31**. Cyclic voltammogram of **[Ir(3-MeOppy)$_2$(bpy)](PF$_6$) 1a** recorded at 298 K at 50 mV/s in MeCN with 0.1 M (*n*Bu$_4$N)PF$_6$

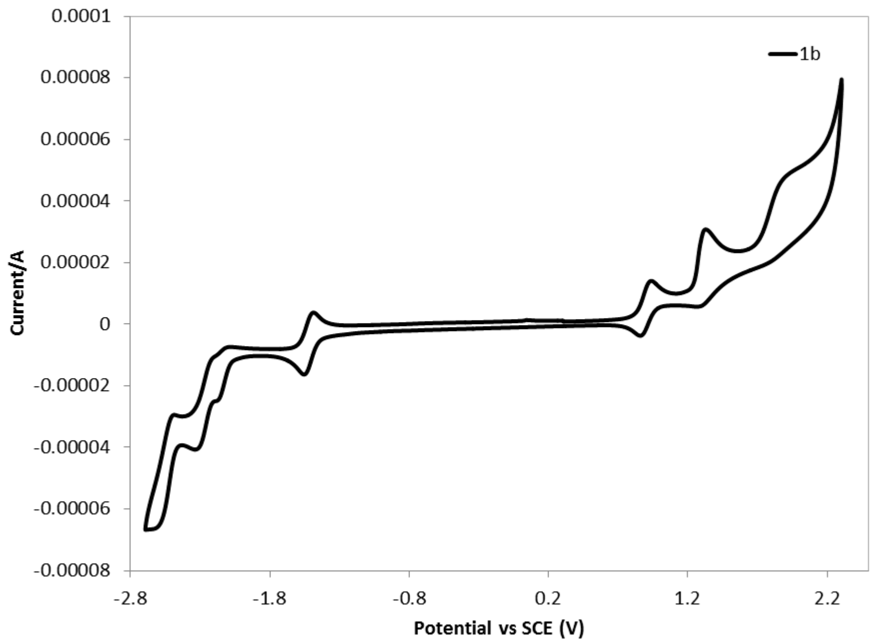

Figure **S32**. Cyclic voltammogram of **[Ir(3-MeO-ppy)$_2$(d*t*bubpy)](PF$_6$) 1b** recorded at 298 K at 50 mV/s in MeCN with 0.1 M (*n*Bu$_4$N)PF$_6$

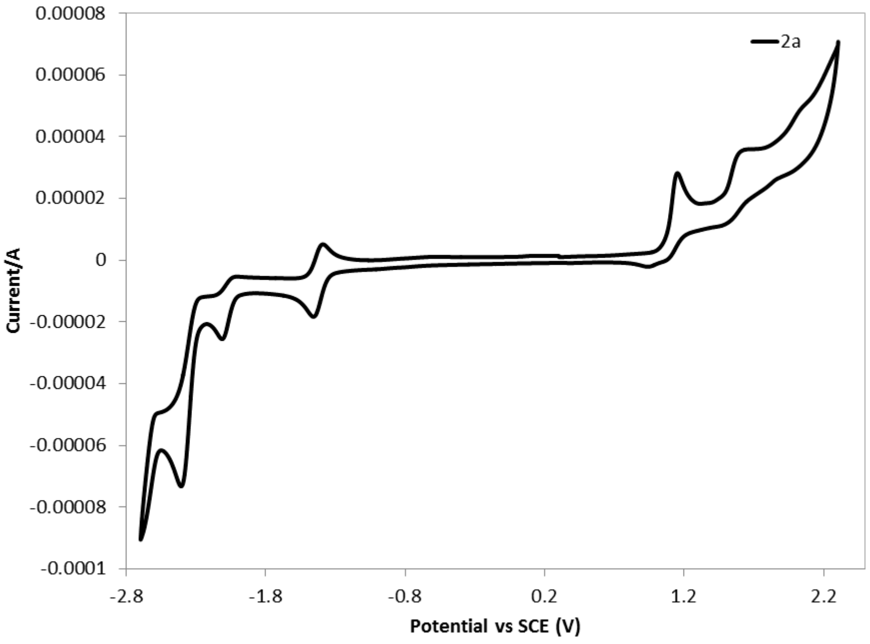

Figure **S33**. Cyclic voltammogram of **[Ir(4-MeOppy)$_2$(bpy)](PF$_6$) 2a** recorded at 298 K at 50 mV/s in MeCN with 0.1 M (nBu$_4$N)PF$_6$

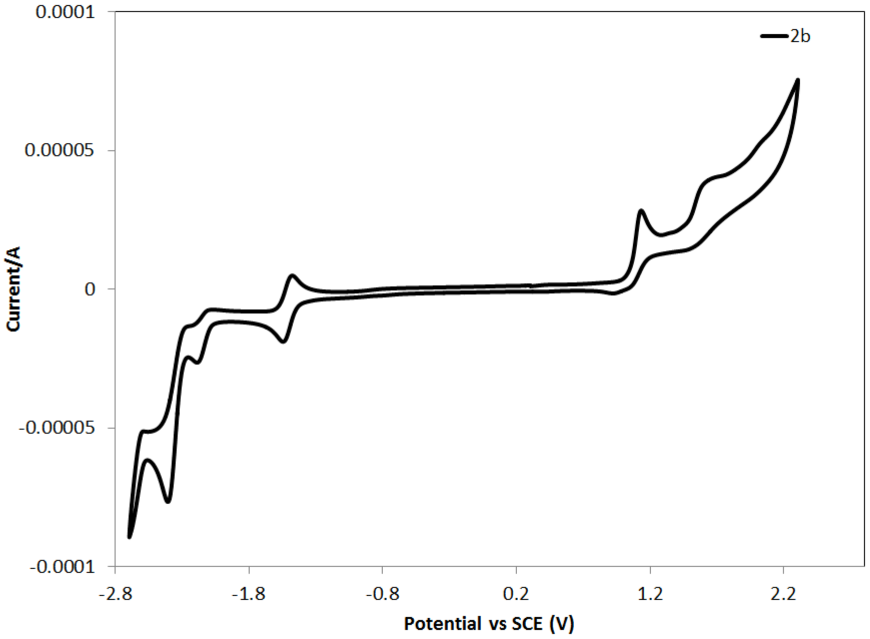

Figure **S34**. Cyclic voltammogram of **[Ir(4-MeOppy)$_2$(d*t*bubpy)](PF$_6$) 2b** recorded at 298 K at 50 mV/s in MeCN with 0.1 M (nBu$_4$N)PF$_6$

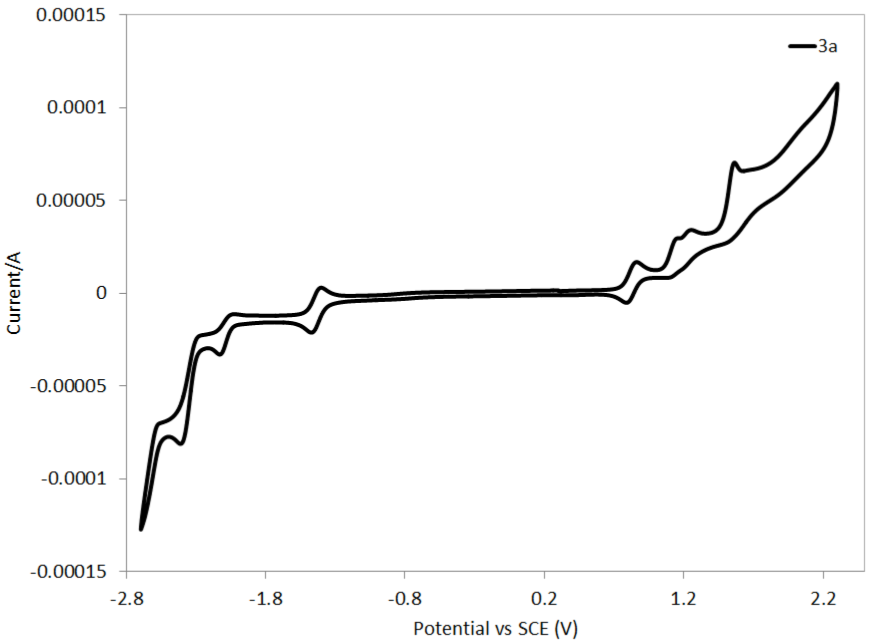

Figure **S35**. Cyclic voltammogram of **[Ir(3,4-dMeOppy)$_2$(bpy)](PF$_6$) 3a** recorded at 298 K at 50 mV/s in MeCN with 0.1 M (nBu$_4$N)PF$_6$

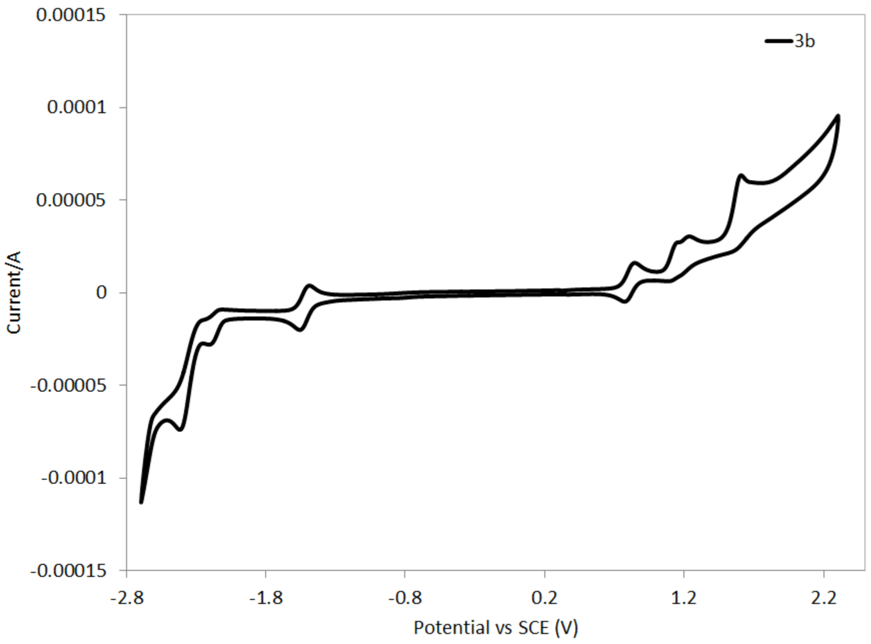

Figure **S36**. Cyclic voltammogram of **[Ir(3,4-dMeOppy)$_2$(d*t*bubpy)](PF$_6$) 3b** recorded at 298 K at 50 mV/s in MeCN with 0.1 M (nBu$_4$N)PF$_6$

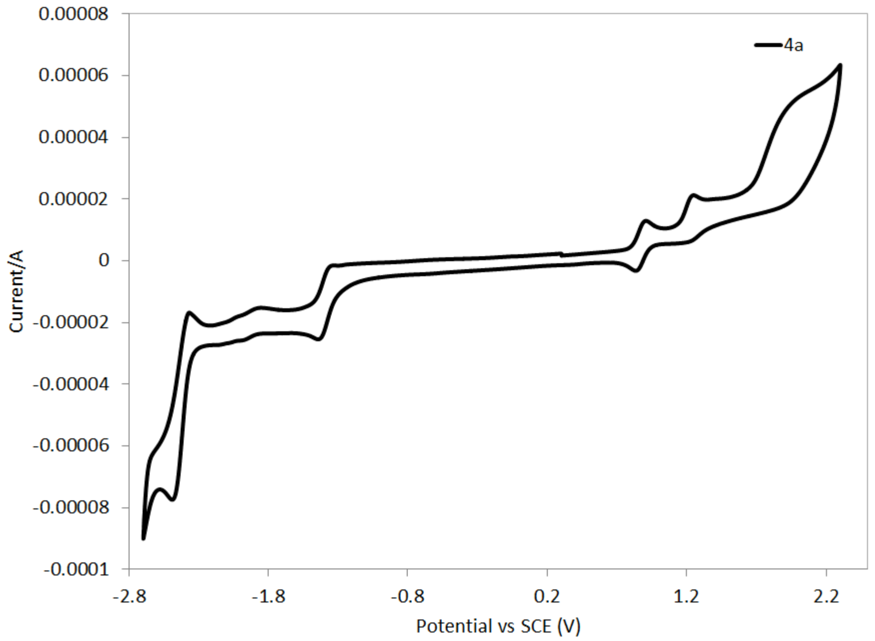

Figure **S37**. Cyclic voltammogram of **[Ir(3,4,5-tMeOppy)$_2$(bpy)](PF$_6$) 4a** recorded at 298 K at 50 mV/s in MeCN with 0.1 M (nBu$_4$N)PF$_6$

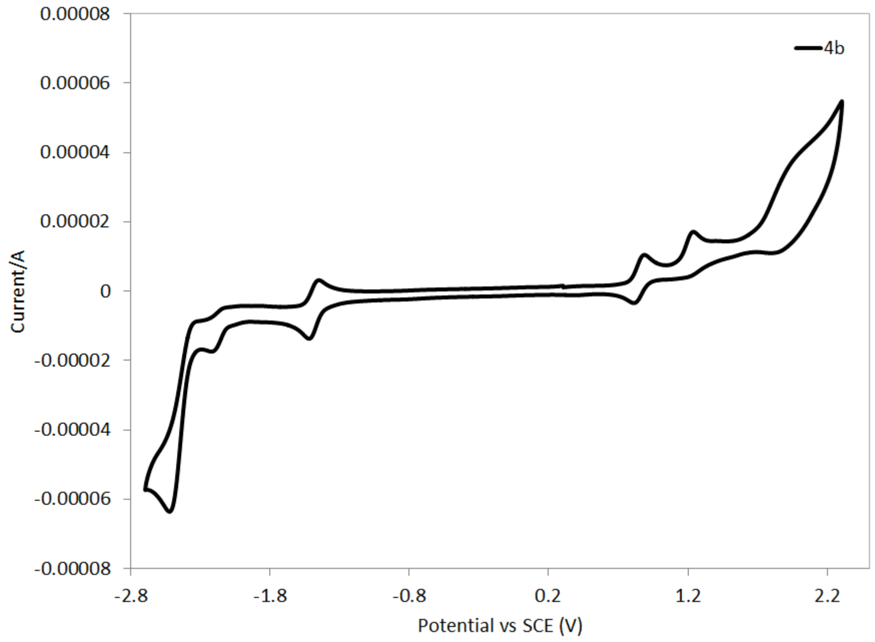

Figure **S38**. Cyclic voltammogram of **[Ir(3,4,5-tMeOppy)$_2$(d*t*bubpy)](PF$_6$) 4b** recorded at 298 K at 50 mV/s in MeCN with 0.1 M (nBu$_4$N)PF$_6$

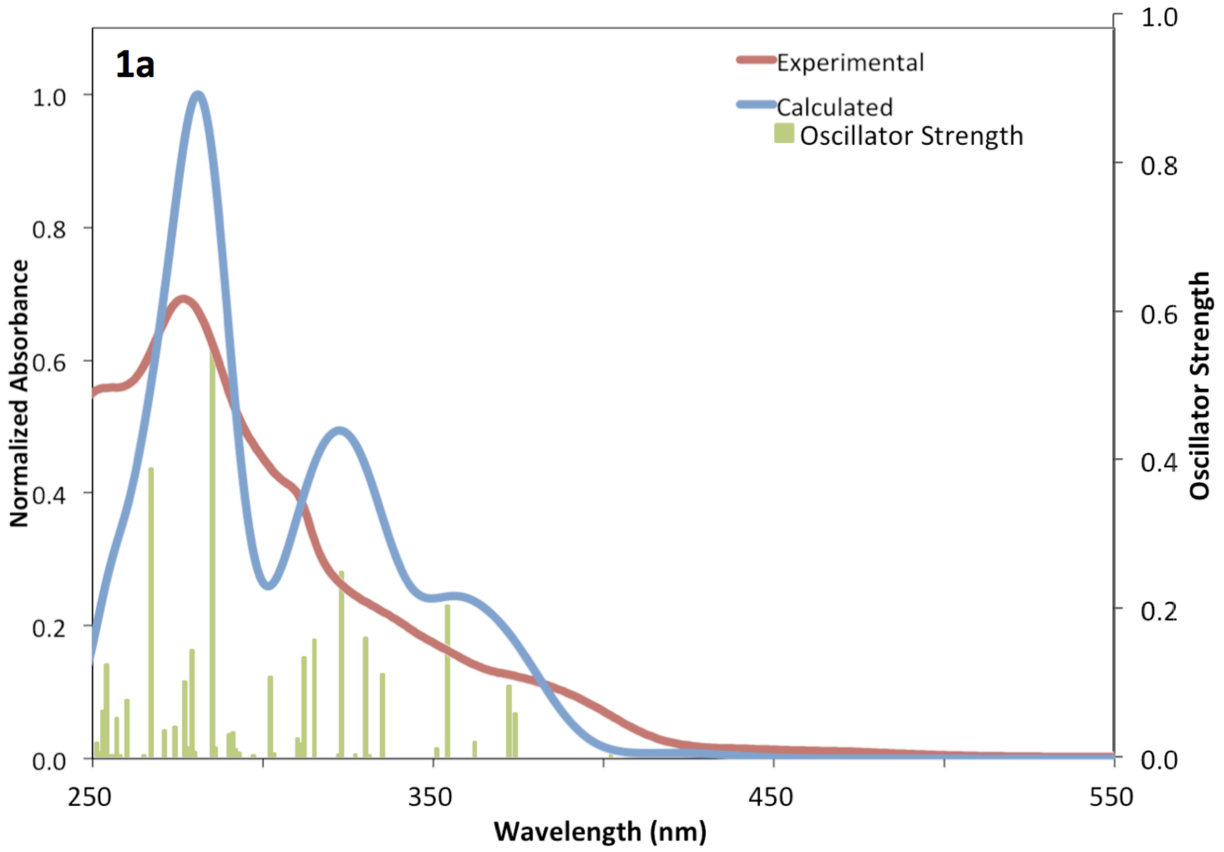

Figure **S39**. Experimental (red) and calculated UV-Vis spectrum (blue – fwhm = 1000 cm^{-1}) obtained from TDDFT calculations with the corresp onding vertical excitations (green) for **1a**.

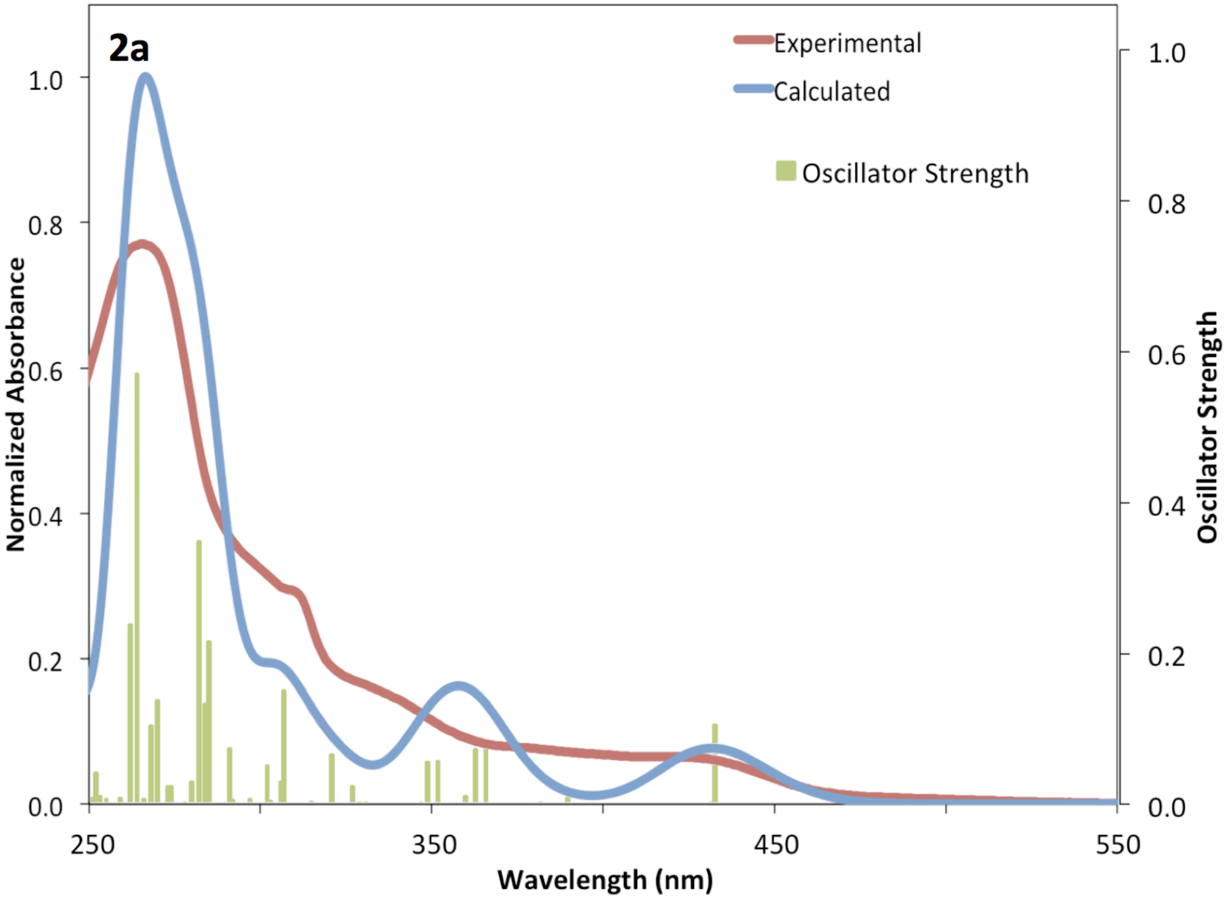

Figure **S40**. Experimental (red) and calculated UV-Vis spectrum (blue – fwhm = 1000 cm^{-1}) obtained from TDDFT calculations with the corresponding vertical excitations (green) for **2a**.

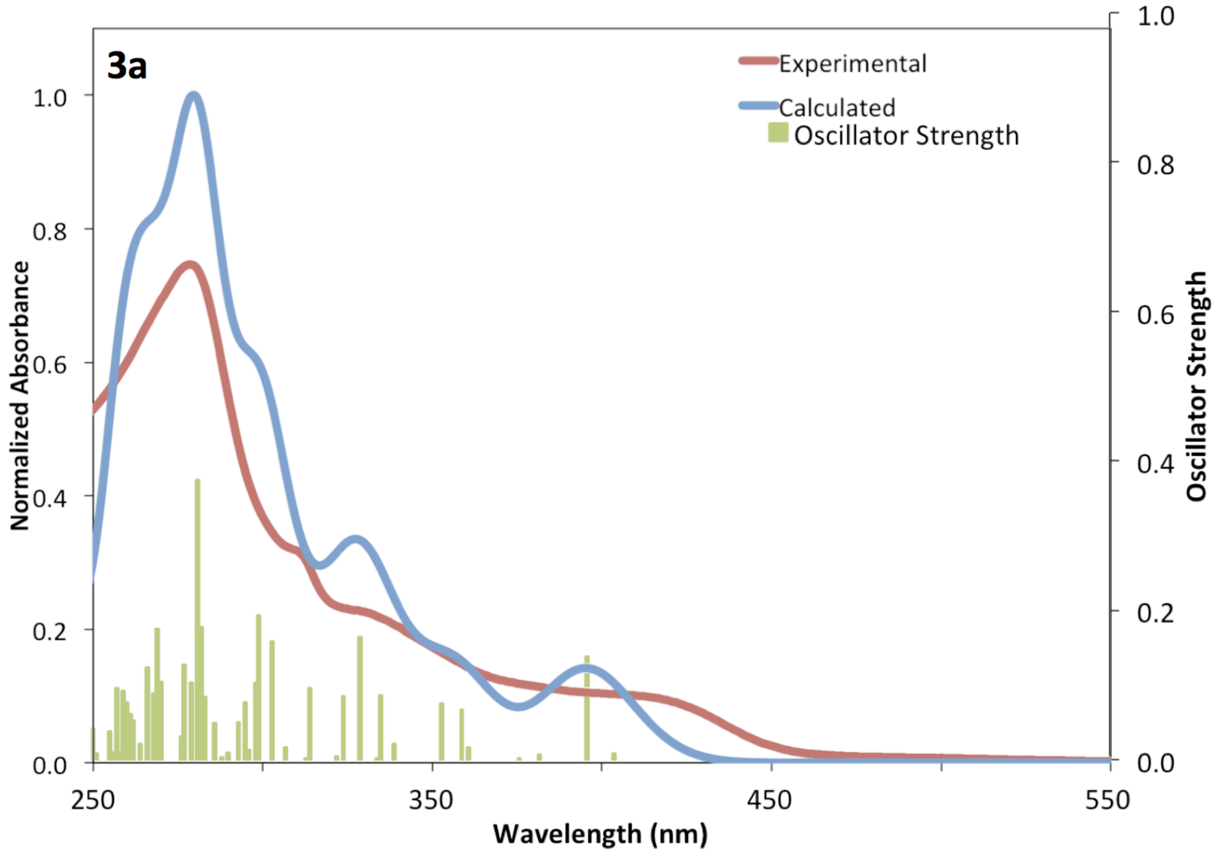

Figure **S41**. Experimental (red) and calculated UV-Vis spectrum (blue – fwhm = 1000 cm^{-1}) obtained from TDDFT calculations with the corresponding vertical excitations (green) for **3a**.

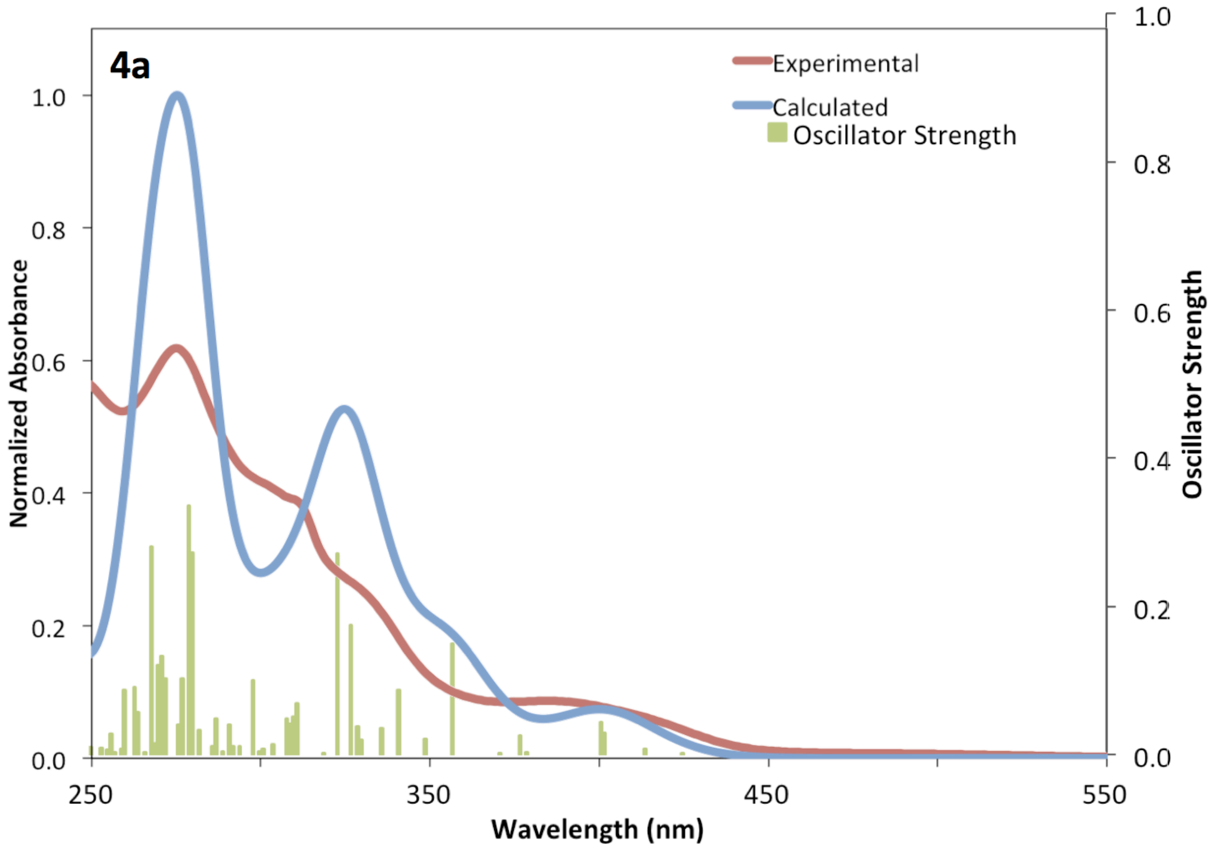

Figure **S42**. Experimental (red) and calculated UV-Vis spectrum (blue – fwhm = 1000 cm^{-1}) obtained from TDDFT calculations with the corresponding vertical excitations (green) for **4a**.

Table S3. Triplet state energies obtained by TDDFT.

	State	λ /nm	E /eV	Assignment [*f*]	Nature
**1a**	T_1	550.7	2.25	H→L (98%) [0]	^{3}MLCT/^{3}LLCT
**1b**	T_1	513.4	2.41	H→L (98%) [0]	^{3}MLCT/^{3}LLCT
**2a**	T_1	483.8	2.56	H→L (91%) [0]	^{3}MLCT/^{3}LLCT
**2b**	T_1	454.7	2.73	H→L (91%) [0]	^{3}MLCT/^{3}LLCT
**3a**	T_1	499.6	2.48	H→L (97%) [0]	^{3}MLCT/^{3}LLCT

**3b**	T_1	469.0	2.64	H→L (97%)	^{3}MLCT/^{3}LLCT
**4a**	T_1	524.1	2.37	H→L (97%)	^{3}MLCT/^{3}LLCT
**4b**	T_1	491.0	2.53	H→L (97%)	^{3}MLCT/^{3}LLCT

The most important parameters for the **1b** - **4b** based LEECs are shown in Table **S4**. The molecule **1a** did not work correctly and no electroluminescence data can be provided.

Table S4. Performance of LEEC devices driven at a constant voltage of 4V.

	t_{max} (s)[a]	$Luminance_{max}$ (cd m^{-2})	$t_{1/2}$ (s)[b]	Efficacy (cd A^{-1})	Power Efficiency (lm W^{-1})	EQE (%)[c]	$CIE_{x,y}$
**1a**	-	-	-	-	-	-	-
**1b**	17	7.4	60	6.7 10^{-4}	0.037	0.0005	0.56, 0.44
**2a**	3	53.1	30	38 10^{-4}	0.0144	0.002	0.49, 0.50
**2b**	7	4.6	42.7	6.3 10^{-4}	0.0004	0.0003	0.46, 0.52
**3a**	30	3.3	100	4.5 10^{-4}	0.0002	0.0004	0.56, 0.44
**3b**	14	7.34	233	8.6 10^{-4}	0.0005	0.001	0.57, 0.43
**4a**	8h	21	16h	0.029	0.007	0.034	0.63, 0.37
**4b**	1.1h	18	2h	0.050	0.013	0.050	0.61, 0.38

[a] Defined as the time to reach the peak luminance. [b] Time to reach half of the maximum luminance. [c] External Quantum Efficiency.

References

1 Pavlishchuk, V. V. & Addison, A. W. Conversion constants for redox potentials measured versus different reference electrodes in acetonitrile solutions at 25°C. *Inorg. Chim. Acta* **298**, 97-102, doi:10.1016/s0020-1693(99)00407-7 (2000).
